# CRISPR/Cas9-mediated disruption of *OsPLDβ1* enhances salt stress tolerance in rice through modulation of antioxidant defense

**DOI:** 10.3389/fgeed.2026.1904040

**Published:** 2026-09-21

**Authors:** Sangeetha Karippadakam, V. Mohan M. Achary, Dalia Vishnudasan, Hemangini Parmar, Alexander Balamurugan, Mallesham Bulle, Ganesan Prakash, Malireddy K. Reddy

**Affiliations:** 1 Crop Improvement Group, International Centre for Genetic Engineering and Biotechnology, New Delhi, India; 2 School of Biotechnology, Amrita Vishwa Vidyapeetham, Kollam, Kerala, India; 3 ICAR- Indian Agricultural Research Institute (IARI), Plant Pathology, New Delhi, India; 4 School of Plant, Environmental and Soil Science, Louisiana State University Agricultural Center, Baton Rouge, LA, United States

**Keywords:** *OsPLDβ1*, CRISPR/Cas9, rice, salt stress, salinity tolerance, antioxidant defense, reactive oxygen species

## Abstract

Soil salinity is a critical abiotic stress that severely constrains rice productivity and threatens global food security. Phospholipase D (PLD) enzymes play pivotal roles in plant stress signaling; however, the specific function of *OsPLDβ1* in rice salt stress responses remains unclear. We here investigated the role of *OsPLDβ1* using knockout mutants to elucidate its regulatory function under saline conditions. *Ospldβ1*-mutant lines exhibited substantially improved salt tolerance in contrast to wild-type plants. Improved tolerance was associated with elevated reactive oxygen species (ROS) scavenging capacity, indicating improved oxidative stress management. Furthermore, the mutant lines displayed increased expression of ion transport and homeostasis genes and maintained photosynthetic efficiency and agronomic performance. Collectively, these physiological and molecular responses demonstrate that *OsPLDβ1* negatively regulates salt stress tolerance in rice. This research highlights the role of *OsPLDβ1* in salt tolerance in rice and lays the groundwork for developing salinity-resistant crop varieties through genetic manipulation.

## 1 Introduction

Salt stress is a significant factor contributing to substantial reductions in global agricultural productivity due to its detrimental effects on plant growth (Behera et al., 2022). The anticipated expansion in the salinization of agricultural land is expected to have significant consequences worldwide, leading to a reduction in land availability by approximately 30% over the course of the next 25 years, with a potential increase of up to 50% by the year 2050 (Wang et al., 2003). Salt stress in plants leads to various morphological and physiological changes as a result of modifications in gene expression and signaling pathways. Plants commonly adopt a transcriptional reprogramming strategy for activation of stress-responsive genes in response to environmental stress conditions (Tolosa and Zhang, 2020). While the traditional breeding method has made significant contributions to enhancing the ability of crops to withstand abiotic stress, it is necessary to adopt novel technologies in order to achieve the anticipated improvements in crop yields. These technologies will enable rapid growth and deployment of future crop varieties, including features enabling them to adapt to stressful environmental conditions. Moreover, agricultural researchers may take longer to respond to the emerging requirements of farmers, producers, and the industry due to the lengthy traditional breeding process for stress-tolerant cultivars (Manavalan et al., 2009). Therefore, additional latest technologies with rapid impacts are needed to deal with these challenges. Genome editing techniques provide precise modification of an organism’s DNA by generating targeted mutations such as insertion or deletion (indel) and by deliberately altering specific DNA sequences using specific nucleases. In recent years, various techniques for genome editing have been developed and employed, including meganucleases (Puchta et al., 1993), transcription activator-like nucleases (TALENs) (Zhang et al., 2013), zinc-finger nucleases (ZFNs) (Zhang et al., 2010), and the recent CRISPR/Cas9 system (Jiang et al., 2013). Several genes have been identified and characterized through CRISPR-/Cas-based genome editing to improve plant salt tolerance in rice. Knockout of negative regulators such as *OsDST* (Santosh Kumar et al., 2020), *OsRR2* (Zhang A. et al., 2019), *OsPQT3* (Alfatih et al., 2020), and *OsSPL10* (Lan et al., 2019) genes in *Oryza sativa* plants using CRISPR/Cas9 resulted in significant tolerance toward salt stress.

Plant phospholipase D (PLD) genes play a pivotal role during plant growth and in various stresses such as salt, drought, and cold (Wang, 2005). The PLD pathway plays a significant role in facilitating the rapid accumulation of phosphatidic acid (PA) in response to salt stress. According to the previous report, *PLDα1, PLDα3,* and *PLDδ* have been implicated in the response to salt stress. Mutations in these genes have been observed to result in decreased levels of PA, elevated sodium ion (Na^+^) levels, and increased susceptibility to salt (Yu et al., 2010; Hong et al., 2008; Bargmann et al., 2008). Previous data suggest that *PLDα1-*produced PA activates MPK6. Additionally, it phosphorylates the C-terminus of SOS1, resulting in the removal of excessive Na^+^ from *Arabidopsis* plant cells (Yu et al., 2010). Additional research has revealed that PA generated from *PLDα1* has the ability to bind and activate the upstream components of *MPK6*, namely, *MKK7* and *MKK9*. This leads to the formation of a cascade involving *MKK7/MKK9* and *MPK6*, which ultimately results in the phosphorylation of SOS1 and removal of excessive Na^+^ from plant cells (Shen et al., 2019). The soybean MAPK, known as GMK1, exhibits stimulation in response to PA during salt stress (Im et al., 2012). This finding demonstrates that regulation of the MAPK cascade signaling system and SOS pathway in plants can be enhanced through the involvement of PLD-derived PA, leading to improved salt stress tolerance. Salt stress in rice suspension-cultured cells leads to enhanced *PLDα* activity and protein localization in the plasma membrane and tonoplast. Studies on knockdown lines of *PLDα* suggest that it regulates rice salt stress response by modulating PM H + -ATPase activity and transcription levels of OsNHX1 (TP Na^+^/H^+^ antiporter), OSA2 (PM H + -ATPase) and OsVHA-A (tonoplast H + -ATPase) (Shen et al., 2010). The expression levels of jute PLD genes, specifically *CcPLDα1-1*, *CcPLDα1-2*, *CcPLDα2*, *CcPLDγ1*, and *CcPLDδ2*, were found to be more abundant in *C. capsularis* than in *C. olitorius* while exposed to salt and drought stress. This finding suggests that PLD genes play a significant role in abiotic stress tolerance in *C. capsularis* (Sadat et al., 2022). The gene *AnPLDα* shows elevated expression in response to high salt conditions in the xerophyte *Ammopiptanthus nanus*. Furthermore, when it is introduced into *Arabidopsis*, the heterologous expression of *AnPLDα* has been shown to enhance the ability to tolerate high salt stress conditions (Yu et al., 2015). The salt tolerance of *Nicotiana tabacum* is enhanced through the heterologous expression of a *CsPLDα* by regulating the balance of Na^+^ and K^+^ ions, reducing lipid peroxidation, and facilitating the accumulation of osmoprotective substances such as proline, soluble carbohydrates, and soluble proteins (Ji et al., 2017). In tomato, *LePLDα1* contributes to salt-induced PA production, although its reduction does not significantly alter salt tolerance. Conversely, *Arabidopsis AtPLDα*1 and *AtPLDδ* are essential for stress resilience; their deficiency leads to hypersensitivity and stunted growth under salinity and hyperosmotic stress. Collectively, these results identify PLD as a key, yet species-variable, mediator of plant responses to dehydration (Bargmann et al., 2008). Dual overexpression of PeNADP-ME and *PePLDδ* in *Arabidopsis* significantly boosts salt resilience through synergistic PA signaling. This interaction enhances Na^+^ exclusion and ROS scavenging via increased antioxidant activity (SOD, APX, and POD), demonstrating that amplified PLD-derived PA signaling is a key driver of ionic and redox homeostasis under salinity (Zhang et al., 2024).

These findings suggest that PLD-derived PA has a broad function in salt stress response. This investigation examines the role of *OsPLDβ1* in high salinity stress, employing CRISPR-/Cas9-mediated knockout in an indica rice cultivar. We demonstrate that in both vegetative and reproductive phases, the lack of *OsPLDβ1* leads to increased tolerance in high salt-containing medium. Manipulation of the ortholog genes could facilitate the development of salt-tolerant cultivars adapted to saline-prone environments, thereby supporting sustainable agricultural productivity. However, further studies are required to elucidate the underlying molecular mechanisms and validate its potential application in crop improvement.

## 2 Materials and methods

### 2.1 Plant materials

The Indica BPT5204 rice variety obtained from ICAR-IIRR, Hyderabad, was chosen to develop CRISPR/Cas9 knockout lines to investigate the function of the *OsPLDβ1* locus. *Ospldβ1-1 and Ospldβ1-2* mutant homozygous T3 generation seeds developed previously in our crop improvement lab (Karippadakam et al., 2026) were used for all the salt stress assays (Supplementary Figure S1).

### 2.2 Development and validation of CRISPR-/Cas9-mediated *Ospldβ1* mutants

The CRISPR-/Cas9-mediated *OsPLDβ1* knockout lines used in this study were previously generated and characterized by Karippadakam et al. (2026). Briefly, a gene-specific sgRNA (CAG​CAG​TAT​CCT​TAC​CCG​TAC​GG) targeting *OsPLDβ1* was designed using CRISPR-direct and cloned into the pMDC99 binary vector containing the eSpCas9 expression cassette. Rice transformation was performed via *Agrobacterium*-mediated plant transformation. The putative plants developed through tissue culture were screened for Cas9 integration using polymerase chain reaction (PCR). PCR-positive plants were selected for Sanger sequencing to confirm targeted mutations. Homozygous mutant lines were identified by sequence alignment with the wild-type plant. The effects of the mutations on the coding sequence were predicted using the ExPASy Translate tool (Supplementary Figures S2A,B). Additionally, qRT-PCR analysis of *OsPLDβ1* transcript levels was used to validate the functional knockouts. T-DNA-free homozygous mutant lines were identified by PCR-based segregation analysis of the *Cas9* and *hpt* genes and were used for all subsequent experiments. Detailed information on sgRNA design, vector construction, mutation identification, and validation of mutants has been reported previously (Karippadakam et al., 2026).

### 2.3 Chlorophyll retention assay

Fully expanded leaves from the vegetative stage of WT and *Ospldβ1* mutant plants were used to measure chlorophyll retention under salt stress conditions. Leaves were cut into small pieces (1–2 cm) and floated on distilled water (Control) and exposed to 100 mM, 150 mM, and 200 mM NaCl in a Petri plate, followed by incubation in a growth chamber room under controlled conditions of 16-h/8-h light/dark cycle at 28 °C for 4 days. The photographs were taken at the end of the experiment, and the dimethyl sulfoxide (DMSO) method by Hiscox and Israelstam (1979) was used for detection of chlorophyll content.

### 2.4 Salt germination assay

To test the growth rate under salt stress conditions, 50 surface-sterilized seeds (dehusked) from each WT and *Ospldβ1* mutant line were placed on half-strength MS agar medium containing varying concentrations of NaCl (0 mM, 100 mM, and 150 mM). The culture medium boxes were placed in a growth chamber under standardized environmental conditions (16-h/8-h light/dark cycle) at 28 °C. The germination percentage and root length and shoot length were measured at the 10th day of salt treatment. All the seed germination assay experiments were repeated three times with the same laboratory conditions.

### 2.5 Salt stress assay at the vegetative stage

To evaluate salt stress tolerance during the vegetative stage, WT and *Ospldβ1* mutant seeds were surface-sterilized with Bavistin (0.5%) for 5 min. Bavistin residues were removed by washing with sterile water for 5 min. A total of 40 seeds from each line were germinated and grown on Yoshida medium in a plastic box for 14 days under greenhouse conditions (relative humidity: 70%–80%; temperature: ±28 °C). Salt stress was induced with 150 mM NaCl along with Yoshida medium on the 14th day. After 7 days of salt stress, the planting box was replaced with fresh Yoshida nutrition solution for recovery. The percentage of survival was determined on the 5th day post-recovery. Root number and root length were measured at the 7th day of salt stress treatment. A scanner is used to take images of the root morphology. The physiological and biochemical changes were monitored on the 2nd day of salt treatment to evaluate the early responses of plants to salt stress, before the development of severe stress-induced damage.

For evaluating the salt stress at the early maturation stage, surface-sterilized seeds were germinated and maintained under greenhouse conditions for 14 days in a plastic box with nutrient Yoshida medium. WT and *Ospldβ1* mutant seedlings were transferred into plastic pots filled with a mixture of soil and fertilizer and grown for 2 months. All plants were irrigated twice a week. After 2 months, salt stress was introduced into the irrigation program by adding 150 mM NaCl in the irrigation water. The treatment is continued till all WT lines were completely dried, and then the phenotype was compared with that of *Ospldβ1* mutant lines.

### 2.6 Histochemical and biochemical analyses

Using the technique of Achary et al. (2012), histochemical studies of two major ROS molecules, O_2_^•−^ superoxide (O_2_^•−^) and hydrogen peroxide (H_2_O_2_), were performed. Superoxide (O_2_•^-^) accumulation was histochemically visualized in detached leaves using nitro blue tetrazolium (NBT) staining. The leaves were immersed in a solution containing 50 mM Tris–HCl buffer (pH 6.4), 0.2 mM NBT, 0.2 mM NADH, and 250 mM sucrose. The samples were vacuum-infiltrated for 15 min and subsequently incubated under light for 48 h. H_2_O_2_ accumulation was histochemically visualized in detached leaves using 3,3′-diaminobenzidine tetrahydrochloride hydrate (DAB) staining. The leaves were immersed in 10 mM sodium phosphate buffer containing 1 mg mL^-1^ DAB (pH 4.5) and vacuum-infiltrated for 15 min. The leaves were then incubated overnight at room temperature in the dark and subsequently exposed to light until brown patches developed. The leaves were bleached using 95% ethanol at 90–95 °C for 15 min in a water bath to remove chlorophyll and facilitate visualization of ROS molecules. The experiment was repeated three times with nine biological replicates.

For estimation of the H_2_O_2_ level, 0.5 g of the leaf tissue was homogenized with 3 mL of 0.1% (w/v) trichloroacetic acid, followed by centrifugation at 10,000 *g* for 20 min. The supernatant (0.5 mL) was added to 1 mL of 1 M potassium iodide and 10 mM sodium phosphate buffer (pH 7.0). The reaction mixture was incubated for 5 min. The absorbance was recorded at 390 nm and expressed in µmol g^-1^ FW.

Thiobarbituric acid (TBA) assay was employed to measure the production of malondialdehyde (MDA) using a spectrophotometric method. Approximately 100 mg of the leaf tissue was homogenized with 20% trichloroacetic acid (2 mL) and centrifuged at 8000 *g*. The supernatant (0.5 mL) was added to 2 mL of a reaction mixture containing trichloroacetic acid (20%) and TBA (0.5%), followed by incubation at 95 °C for 30 min. The absorbance was recorded at 532 nm and 600 nm and expressed in µmoles g^-1^ FW. Three independent biological and technical replicates were used for the experiment.

According to Hiscox and Israelstam (1979), DMSO was used to estimate the amount of chlorophyll. Fifty milligrams of leaf tissue was collected from three independent biological and three technical replicates of WT and mutant lines. The leaves were incubated with 10 mL of DMSO in the dark overnight, followed by heating at 65 °C for 4 h in dark conditions. The absorbance was recorded at 663 and 645 nm using a Cytiva Ultrospec™ 8000 UV–Visible spectrophotometer, taking DMSO as blank. The experiment was repeated three times, and chlorophyll pigments were quantified using the formula:

$$\text{Total chlorophyll }\left(\text{mg }g^{-1}\text{ FW}\right)=\left[\left(20.2\;\times {\text{OD}}_{645}\right)+\left(8.02\;\times {\text{OD}}_{663}\right)\right]\times V/\left(1000\;\times \;W\right).$$

The Horiba LAQUAtwin EC-33 Compact Conductivity Meter was used to estimate electrolyte leakage. Approximately 100 mg of the leaf tissue was collected from WT and mutant plants, with three independent biological replicates and technical replicates for each sample. The leaf tissues were immersed in 10 mL of Milli-Q water and incubated at 40 °C for 30 min in a water bath. The electrical conductivity (C1) of the solution was then measured using a conductivity meter. The same samples were subsequently incubated at 100 °C in a boiling water bath for 10 min, after which the electrical conductivity (C2) was measured. Electrolyte leakage was calculated using the following formula: Electrolyte leakage (%) = (C1/C2) × 100.

Measurement of free proline content was measured before and after the salt treatment, followed by few modifications (Bates et al., 1973). Free proline was calculated according to the formula 36.6 × A520 × volume/2× FW and expressed in μmol g^-1^ FW. To measure the H_2_O_2_ level in plants, 300 mg of leaf tissue was subjected to homogenization in 3 mL trichloroacetic acid (0.1%w/v) and centrifuged at 12,000 *g* for 20 min, and then 0.5 mL of the supernatant (leaf extract) was mixed with 1 mL potassium iodide (1 M) and 1 mL potassium phosphate buffer (10 mM) at pH 7.0 and incubated for 5 min. The absorbance was measured at 390 nm and expressed in nmolg^-1^ FW using a H_2_O_2_ standard curve. All the biochemical analyses were performed on the second day of salt treatment to evaluate the early responses of the plants to salt stress, before the development of severe stress-induced damage.

### 2.7 Spectrophotometric determination of antioxidant enzymatic activities of SOD, APX, and CAT

Achary et al. (2012) measured the concentrations of antioxidant enzymes catalase, superoxide dismutase, and ascorbate peroxidase. Leaf samples from WT and mutants were collected in control and salt stress conditions from three independent biological and technical replicates. To isolate the total protein content, 1 g leaf tissue was ground with liquid nitrogen using a mortar and pestle, followed by homogenization with 2 mL of 50 mM Tris–HCl buffer, pH 7.2, containing 0.1 mM ethylenediamine-tetra acetic acid (EDTA) and 1% (w/v) polyvinyl-polypyrrolidone at 4 °C. Additionally, for the ascorbate peroxidase assay, a homogenization buffer containing 5 mM ascorbate was used. The homogenization was followed by centrifugation for 15 min at 10,000 *g* at 4 °C. The soluble protein concentration was measured using the Bradford method (1976) with bovine serum albumin as the reference.

Catalase (CAT) activity assay was conducted based on the decomposition of H_2_O_2_, which was monitored by measuring the decrease in the absorbance at 240 nm: 10 mM of H_2_O_2_ was mixed with 50 mM sodium phosphate buffer (pH 7.0), followed by the addition of the enzyme extract containing 50 µg of total soluble protein in a final reaction volume of 1 mL. The decrease in the absorbance at 240 nm was monitored spectrophotometrically for 2 min at 30-s intervals. The rate of H_2_O_2_ decomposition was calculated using an extinction coefficient of 39.4 mM^-1^ cm^-1^. CAT activity was normalized to total soluble protein concentration and expressed as µmol H_2_O_2_ min^-1^ mg^-1^ protein.

Superoxide dismutase (SOD) activity was monitored by inhibiting the photochemical reduction of NBT. The 3-mL reaction mixture contained 50 mM potassium phosphate buffer (pH 7.8), 13 mM methionine, 75 μM NBT, 2 μM riboflavin, 0.3% (v/v) Triton X-100, 0.1 mM EDTA, and 50 μg protein extract. The reaction was commenced by light illumination (Phillips 40 W x 2 fluorescent) for 8 min at room temperature, and the absorbance was recorded at 560 nm. One unit of SOD activity was defined as the amount of the enzyme required to inhibit the photochemical reduction of NBT by 50% and expressed as U mg^-1^ total soluble protein.

APX activity was determined using 3 mL of the reaction mixture composed of 50 mM sodium phosphate buffer (pH 7.0), 0.5 mM ascorbic acid, and 0.2 mM H_2_O_2_ and 50 μg of the protein extract. Oxidation of the ascorbate was estimated by measuring the decrease in the absorbance at 290 nm (Σ = 2.8 mM^-1^ cm^-1^). APX activity was expressed as µmol ascorbate oxidized min^-1^ mg^-1^ total soluble protein.

### 2.8 Plant gas exchange and photosynthesis analysis

The portable infrared gas analyzer (LICOR 6400) was used to measure the photosynthetic parameters stomatal conductance (gs), transpiration rate (E), intercellular CO_2_ concentration (Ci), and photosynthetic rate (A). Measurements were taken from the fully expanded second uppermost leaf of 2-month-old plants, using five independent WT and *Ospldβ1* plants. The reference CO_2_ was maintained at 400 ppm, flow rate at 300 μmol s^-1,^ and the light intensity was maintained at 1,000 μmol m^-2^ s^-1^ of photosynthetically active radiation (PAR). Leaf temperature was maintained at 28 °C, and the leaf vapor pressure deficit (VPD) ranged from 2.2 to 3.5 kPa. Measurements were performed between 10:00 a.m. and 12:00 p.m. using a 2-cm^2^ leaf chamber.

### 2.9 Plant RNA isolation and real-time PCR

Leaf samples were collected from the WT and *Ospldβ1* mutant lines on the second day after salt treatment. The harvested sample was placed in liquid nitrogen immediately after collection. The TRIzol-based method was used to extract high-quality RNA from leaf tissues and purify them. The concentration and purity of the RNA were determined using a NanoDrop instrument by measuring the A260/A280 ratio. RNA integrity was further verified by agarose gel electrophoresis. The Verso cDNA Synthesis Kit (Thermo Scientific) is used to produce cDNA from 1 μg of RNA. Using the SYBR Green-based PCR assay, quantitative real-time polymerase chain reaction (RT-qPCR) was carried out under the Real-Time PCR System. Each qRT-PCR analysis was performed using three independent biological replicates, with each biological replicate analyzed in three technical replicates. The procedure for RT-PCR as follows: 1 cycle for 10 min at 95 °C, 40 cycles for 15 s at 95 °C, and 1 min at 60 °C. A melt-curve analysis was performed to confirm amplification specificity, and all primer pairs produced a single sharp peak without detectable primer–dimer formation or nonspecific amplification. The 2^−ΔΔCT^ method was utilized to analyze the RT-qPCR result, with the rice ubiquitin gene (*RUBIQ1*) serving as a reference. The RT-qPCR primer sequences and the corresponding raw data are provided in Supplementary Tables S1, S2, respectively.

### 2.10 Salt stress treatment at maturation and agronomic performance analysis

For salt stress study during the maturation stage, surface-sterilized seeds were germinated and grown in Yoshida media for 14 days under greenhouse conditions (70%–80% relative humidity at ±28 °C). Seedlings from WT and *Ospldβ1* mutant lines transferred into plastic pots filled with a mixture of soil and fertilizer and grown for 3 months before exposure to salt stress treatments. To induce salt stress, 3-month-old plants were irrigated with 100 mM NaCl solution twice a week, and yield data (tiller number, panicle number, flag leaf length, and no. of grains per panicle) were recorded after 50 days of salt treatment. Photographs of plant were taken at the 50th day of salt treatment and flag leaf at the 15th day of treatment.

### 2.11 Statistical analysis

Biochemical and physiological experiments were conducted in three separate technical replications using multiple biological samples and repeated thrice. The mean values ± standard deviations (STD) are presented in the respective figures. Multiple comparisons were carried out with one-way and two-way analysis of variance (ANOVA) based on Tukey’s test and least significance difference (LSD) at *p* ≤ 0.05 and/or 0.01 levels. GraphPad Prism V8 was used to visualize statistical data. Data significantly increased *p* ≤ 0.05 (a) or 0.01 (b) or significantly decreased *p* ≤ 0.05 (c) or 0.01 (d) from the respective WT plant under control and salt stress conditions. Similarly, data significantly increased or decreased *p* ≤ 0.05 (*) or 0.01 (**) in the same genotypes under control and salt conditions.

## 3 Results

### 3.1 *Ospldβ1*-mutants show better growth under *in vitro* salt stress conditions

Phospholipase D-mediated lipid signaling is integral to plant abiotic stress responses. Two independent *OsPLDβ1* knockout lines were generated previously using the CRISPR/Cas9 system (Figure 1A). The sgRNA was designed to target the first exon of *OsPLDβ1* adjacent to the NGG PAM sequence. Both mutations introduced frameshifts and generated premature stop codons, leading to predicted nonsense-mediated mRNA decay (NMD), which was further validated by qRT-PCR analysis showing significantly reduced *OsPLDβ1* transcript levels in the mutant lines. Although RT-qPCR confirmed the reduced expression of *OsPLDβ1* in knockout lines, off-target mutation analysis and protein-level validation by Western blot were not performed in the study. Following our previous demonstration that disruption of *OsPLDβ1* enhances drought tolerance in rice, the present continuation study investigates the functional role of *OsPLDβ1* under salt stress conditions to determine whether its regulatory effects extend across multiple abiotic stress pathways (Karippadakam et al., 2026). These findings confirm that *OsPLDβ1* functions as a negative regulator of salt stress tolerance and, together with its role in drought response, highlight its potential as a strategic target for developing rice cultivars with broad-spectrum abiotic stress resilience.

**FIGURE 1:**
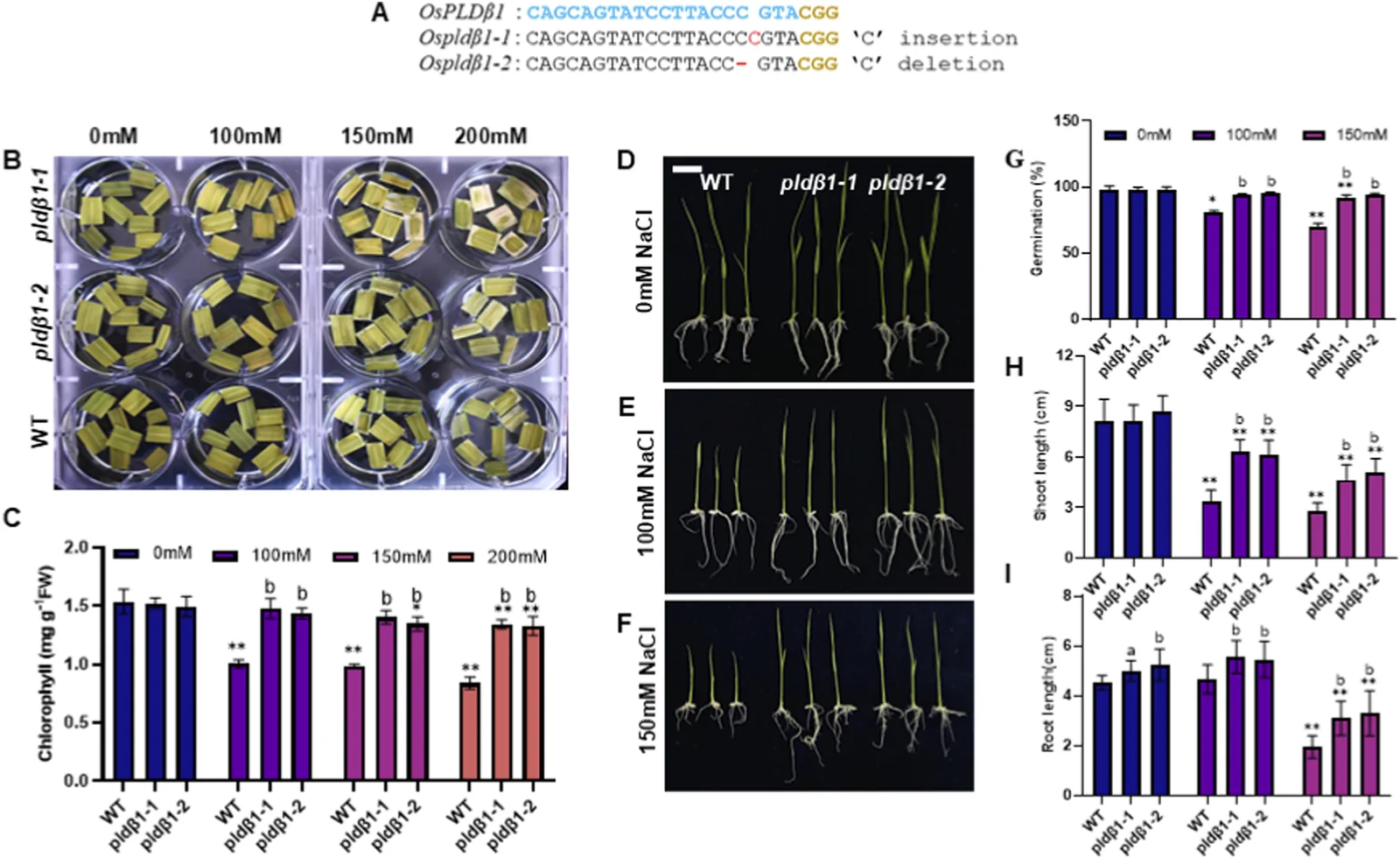
**(A)** The *Ospldβ1-1* and *Ospldβ1-2* lines showed C-insertion and C-deletion mutation. Adapted from the sequence information reported in Karippadakam et al. (2026), licensed under the CC BY 4.0 license. **(B)** Chlorophyll retention assay of *Ospldβ1* mutants. Fully expanded WT, *Ospldβ1-1*, and *Ospldβ1-2* leaves were treated with water (control) and 100 mM, 150 mM, and 200 mM NaCl for 4 days. Photographs were taken, and chlorophyll content was estimated. **(C)** Total leaf chlorophyll content in control and 100 mM, 150 mM, and 200 mM NaCl-treated leaves. Data were recorded in three biological replicates (n = 3). Growth analysis of *Ospldβ1* and WT lines at the seedling stage under salinity treatment. Photographs show representative seedlings germinated and grown on MS medium supplemented with 0 mM, 100 mM, and 150 mM NaCl for 10 days. **(D–F)** The phenotype of WT and mutant lines grown on 0 mM, 100 mM, and 150 mM NaCl-containing medium for 10 days. Germination rate **(G)**, shoot length **(H)**, and root length **(I)** under control and salinity conditions (100 mM NaCl; 150 mM NaCl). Values are expressed as means ± S. D, n = 30. Letters and symbols indicate a significant difference between means (*p* ≤ 0.05 or 0.01, one-way or two-way ANOVA or Tukey’s HSD *post hoc* test). Different letters indicate significant differences between mutant and respective WT plants under the same treatment: (a) increase, p ≤ 0.05; (b) increase, p ≤ 0.01; (c) decrease, p ≤ 0.05; and (d) decrease, p ≤ 0.01. Asterisks indicate significant differences between control and salt stress conditions within the same genotype: *p ≤ 0.05; **p ≤ 0.01.

To evaluate the impact of mutation in the *OsPLDβ1* gene on salinity stress, first we examined chlorophyll retention of the excised leaf under salt stress. Incubation of leaf disks of the wild type (WT), *Ospldβ1-1*, and *Ospldβ1-2* plants on 100, 150, and 200 mM NaCl and water for 96 h showed an early bleaching of the WT leaf compared with *Ospldβ1* mutant plants, as shown in Figure 1B. At all the three different concentrations of NaCl, the reduction in chlorophyll retention was less pronounced in the *Ospldβ1* mutant than in the WT plants. Additionally, we calculated the total chlorophyll content of the *Ospldβ1* mutant and WT leaf disks after 96-h salt treatment. The *Ospldβ1-1* and *Ospldβ1-2* lines exhibited higher chlorophyll content compared with the WT plants (Figure 1C). Salt treatment more quickly affected the total chlorophyll content of WT lines. However, the extent of this decline was much less in the *Ospldβ1* mutant lines (Figure 1B). The preliminary experiment chlorophyll retention assay of *Ospldβ1* mutant plants under salt stress conditions indicates that the *OsPLDβ1* gene knock out may lead to salinity tolerance in rice.

We conducted an *in vitro* seed germination assay to evaluate the germination, root length, and shoot length of mutant lines under salt stress conditions. The seeds from *Ospldβ1* mutant lines and wild-type plants were germinated on half MS media containing 0 mM, 100 mM, and 150 mM NaCl, and shoot/root length was measured following the 10th day post-germination (Figures 1D–F). No obvious difference in the germination rate was observed between WT and *Ospldβ1* mutant lines in 0 mM NaCl plates. However, *Ospldβ1-1* and *Ospldβ1-2* lines showed remarkably higher germination rate during 100 and 150 mM NaCl condition in comparison to the WT lines (Figure 1G). Salt stress severely inhibited growth patterns of WT plants in terms of shoot and root growth, while *Ospldβ1* mutant plants performed relatively better under similar conditions (Figures 1H,I). In order to verify improved salt stress tolerance of the mutant lines, we determined the percentage reduction compared to the control condition. The percentage reduction in shoot length was 58% for the WT, whereas in *Ospldβ1-1* and *Ospldβ1-2* lines, the shoot length percentage reduction was 23% and 29%, respectively, with 100 mM NaCl (Figure 1H). At 100 mM NaCl, the root length was not reduced in any genotype lines compared with the control. However, at 150 mM NaCl, the percentage reduction in root length was 51.2%, 39.0%, and 41.8% in the WT, *Ospldβ1-1*, and *Ospldβ1-2* lines, respectively (Figure 1I). *OsPLDβ1* gene knockout could help in providing the necessary turgor essential for cell growth and adaptation to salt, ultimately leading to better growth and development.

### 3.2 The *Ospldβ1* mutant rice plants are tolerant to salt stress at the vegetative stage

To examine whether *OsPLDβ1* gene knockout conferred resistance to salt stress during the vegetative stage, 14-day-old rice plants from WT and *Ospldβ1* lines were treated with 0 mM NaCl and 150 mM NaCl in the hydroponic culture medium for 7 days (Figures 2A–C). Under control conditions, no obvious differences were observed between any of the tested lines. The leaves of both the WT and *Ospldβ1* mutant rice plants started wilting from the second day of salt treatment, indicating that 150 mM NaCl inhibited their growth. Most of the leaf tip appeared to be bleached and were rolled in WT lines (Figure 2B) after 2 days of salt treatment. This result indicate that the salt stress severely and very quickly affected the WT plants than the *Ospldβ1* mutant plants. On the 7th day of salt treatment, the WT plants indicated severe wilting and drying, while the *Ospldβ1* mutant plants were in a better condition than WT plants (Figure 2C). After the 7 days of salt treatment, plants were transferred to fresh nutrient media without NaCl. After 5 days of recovery, new leaves and roots appeared only on the *Ospldβ1* mutant plants (Figure 2D).

**FIGURE 2:**
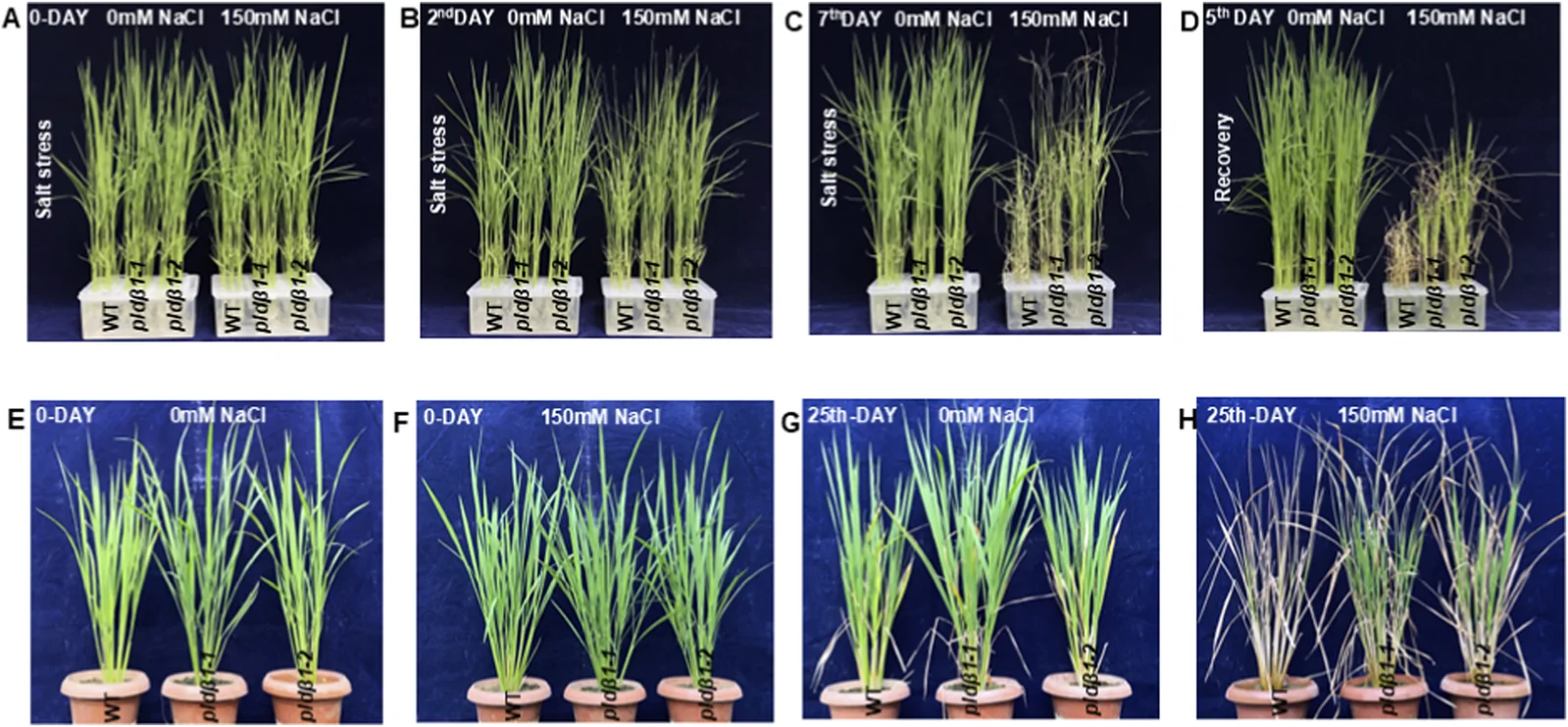
Salt tolerance evaluation of *Ospldβ1* mutants. Two-week-old WT, *Ospldβ1-1,* and *Ospldβ1-2* seedlings grown in Yoshida medium were subjected to salt stress under greenhouse conditions for 7 days **(A–C)**, followed by recovery of seedlings with Yoshida medium for 5 days **(D)**. Two-month-old WT and *Ospldβ1-1* and *Ospldβ1-2* mutant rice plants under salinity stress assay. The growth performance of WT plants and *Ospldβ1* mutants under control conditions **(E,F)** and 25th day of salt treatment **(G,H)**.

In the later stage, we also analyzed the effect of salt stress in 2-month-old WT and *Ospldβ1* mutant rice plants. Before the salt stress treatment, both the WT and *Ospldβ1* mutant plants grew normally (Figures 2E,F). Under control conditions, both WT and Ospldβ1 mutant plants showed normal growth with no obvious differences in phenotype (Figure 2G). On the 10th day of salt treatment, WT plants began to wither, while *Ospldβ1* mutant plants exhibited no obvious indications of stress. WT plants showed severe damage like 95% of drying and rolling after 25 days of salt treatment, while the *Ospldβ1* mutant lines remained green (50%–60%) under severe salt stress (Figure 2H). The results indicated that the mutation of *OsPLDβ1* improved salt stress tolerance in rice lines during the vegetative and seedling stages.

### 3.3 Evaluation of salt stress tolerance of *Ospldβ1* seedlings through phenotypic and physiological analyses

The 2-week-old WT and *Ospldβ1* mutant rice seedlings were maintained in hydroponic solution containing 150 mM NaCl for 7 days (Figure 2A). After a 5-day recovery from salt treatment, 45% of the plants from the *Ospldβ1-1* line and 83% of the plants from the *Ospldβ1-2* line survived, while only 6% of WT plants survived (Figure 3A). Although *Ospldβ1-2* exhibited a higher survival rate than *Ospldβ1-1* during the seedling-stage salt stress assay, both mutant lines displayed significantly improved survival compared with the WT (Figure 3A). The chlorophyll content of *Ospldβ1-1* and *Ospldβ1-2* was significantly higher after the 5th day of salt treatment, while the chlorophyll contents of WT plants were lower (Supplementary Figure S3). As a primary indicator of membrane damage, we measured electrolyte leakage (EL) and malondialdehyde (MDA) level under salt stress. Under control conditions, we could not find significant differences in the electrolyte leakage and MDA level between WT and the *Ospld β1* mutant lines (Figures 3B,C). However, under salinity stress, the *Ospld β1* mutant lines showed a comparatively lower MDA and EL than the WT plants (Figures 3B,C). A rapid increase in proline content with salt treatment was observed in both WT and the *Ospldβ1* mutant lines compared to the control condition. However, the accumulation of salt stress-induced proline was markedly elevated in the leaves of *Ospldβ1* mutant lines compared to WT lines. The proline content at 150 mM NaCl was almost 24% higher in leaves of *Ospldβ1-1* than WT lines, while the proline content was almost 31% higher in *Ospldβ1-2* than the WT lines (Figure 3D). These results suggest that the significantly higher proline accumulation is positively associated with the salt stress tolerance level of the *Ospldβ1* mutant lines.

**FIGURE 3:**
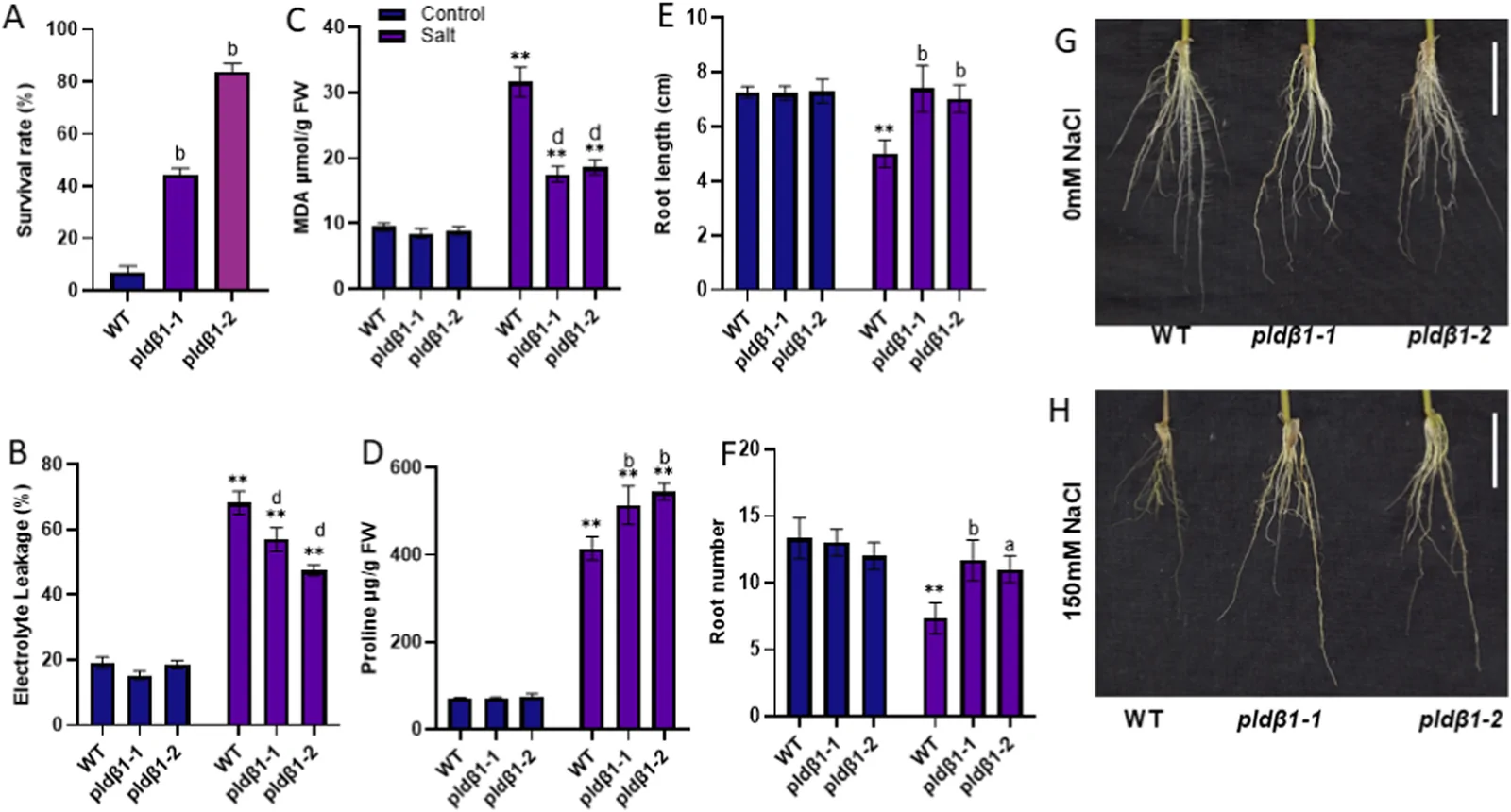
Phenotypic and physiological analysis of *Ospldβ1* mutants rice seedlings during salt stress. **(A)** The survival rate of WT and *Ospldβ1* mutant lines treated with 150 mM NaCl. Data were collected from three different boxes (n = 24 per box). The level of electrolyte leakage **(B)**, MDA **(C)**, and proline **(D)**, during control and salt stress conditions (5th day of stress). Root length **(E)** and root number **(F)** of WT and mutant lines under control and 150-mM NaCl treatment. **(G,H)** Root growth analysis of WT and *Ospldβ1* mutant lines after 7 days of 150 mM salt treatment during the vegetative stage. Scanned image taken after 7 days of salt treatment and control condition. Scale bar = 2 cm. Letters and symbols indicate a significant difference between means (*p* ≤ 0.05 or 0.01, one-way or two-way ANOVA; Tukey’s HSD *post hoc* test). Different letters indicate differences in mutant vs. WT: (a) increase *p* ≤ 0.05, (b) increase *p* ≤ 0.01, (c) decrease *p* ≤ 0.05, and (d) decrease *p* ≤ 0.01. Asterisks indicate differences in control vs. salt stress conditions: *p ≤ 0.05; **p ≤ 0.01.

Additionally, morphological indicators were analyzed to check the salinity response of *Ospldβ1* mutant lines. There were no significant differences in their shoot length, root length, and phenotype under normal conditions (Figure 3G). Under salinity, the shoot length of the *Ospldβ1-1* and *Ospldβ1-2* lines were found to be higher than those of the WT plant (Supplementary Figure S4). *Ospldβ1-1* and *Ospldβ1-2* lines exhibited better root systems characterized by increased length and a greater number of adventitious roots, which were thicker and longer compared to WT plants under 150-mM NaCl treatment (Figures 3E,F,H). Collectively, these results suggest the repressive effect of *OsPLDβ1* on salt tolerance at the seedling stages.

### 3.4 *OsPLDβ1* knockout can tolerate high salinity by maintaining ROS enzymatic homeostasis

Halophytes thrive in high-salinity environments by masterfully balancing reactive oxygen species (ROS) levels. Unlike salt-sensitive plants, they utilize a robust antioxidant defense system through enzymes such as SOD and CAT alongside non-enzymatic compounds to neutralize oxidative stress (Ozgur et al., 2013). ROS can induce irreversible metabolic damage by oxidizing nucleic acids, denaturing proteins, and causing lipid peroxidation (Sharma et al., 2012). To analyze the role of *OsPLDβ1* in ROS scavenging, we measured the contents of two prominent ROS species (H_2_O_2_ and O_2_^.-^) and major enzymatic antioxidant level of mutant plants under salt stress conditions. ROS surge was determined using DAB and NBT leaf staining method under normal and salt stress conditions. DAB staining showed that the WT plants accumulated more H_2_O_2_ than the *Ospldβ1* mutant plants during salt stress (Figure 4A). At the same time, NBT staining displayed lower O_2_^.-^ accumulation in *Ospldβ1* leaves compared with WT plants under salt stress (Figure 4B). These results indicate that loss of function of *OsPLDβ1* may enhance the ROS-scavenging capacity under salt stress in rice plants.

**FIGURE 4:**
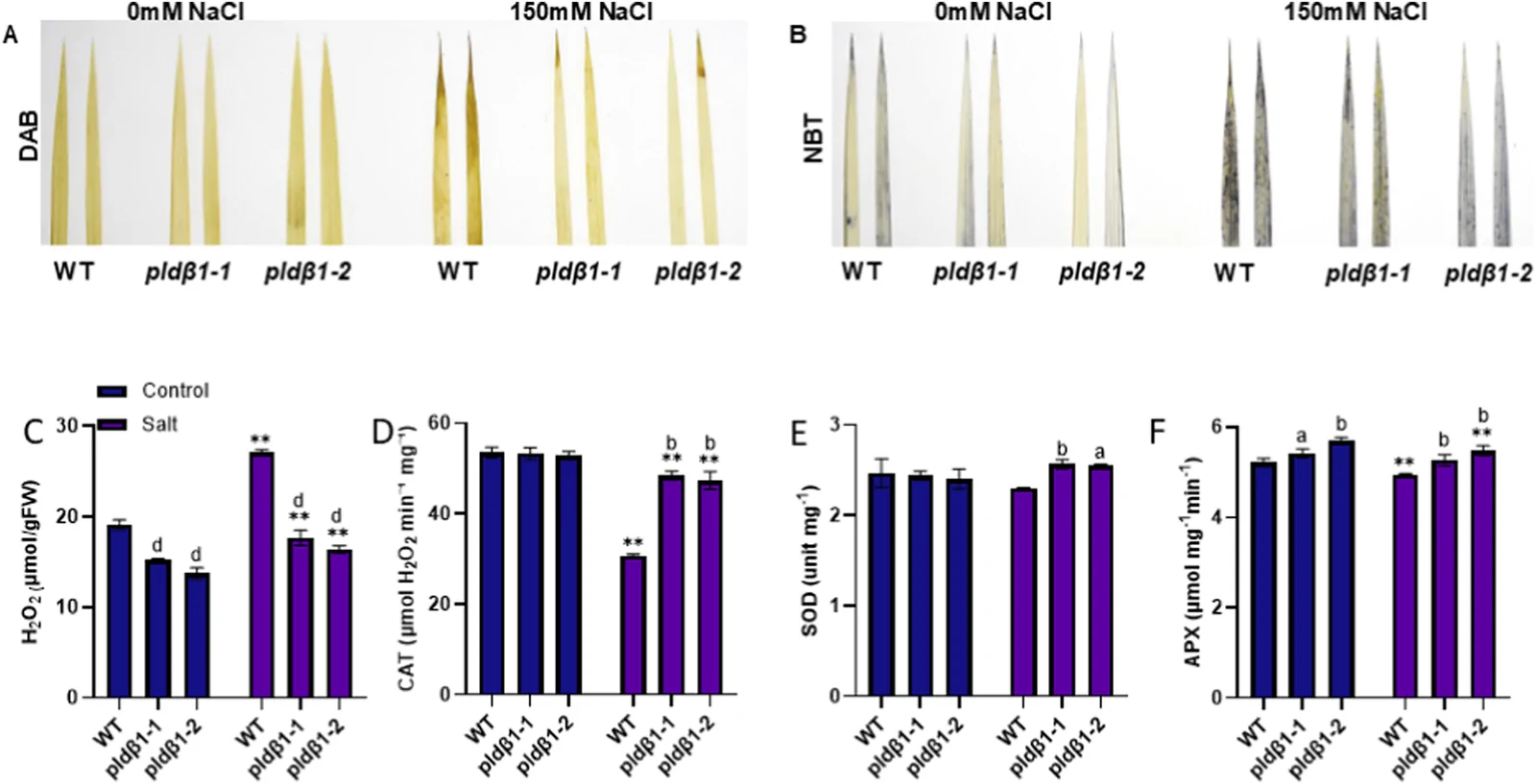
*OsPLDβ1* Knockout enhances tolerance to salt stress by regulating ROS enzymatic homeostasis in rice. **(A,B)** The WT*, Ospldβ1-1*, and *Ospldβ1-2* leaves at the vegetative stage were sampled for DAB and NBT staining for histochemical visualization of H_2_O_2_ and O^2-^, respectively, before and after salt stress (5th day of stress). Levels of H_2_O_2_ **(C)**, CAT **(D)**, SOD **(E)**, and APX **(F)** enzymes before and after salt stress. Letters and symbols indicate a significant difference between means (*p* ≤ 0.05 or 0.01, one-way or two-way ANOVA; Tukey’s HSD *post hoc* test). Different letters indicate mutant vs. WT differences: (a) increase *p* ≤ 0.05, (b) increase *p* ≤ 0.01, (c) decrease *p* ≤ 0.05, and (d) decrease *p* ≤ 0.01. Asterisks indicate differences in control vs. salt stress conditions: *p ≤ 0.05; **p ≤ 0.01.

Further determination of H_2_O_2_ content indicates that the *Ospldβ1* mutant ended up with reduced H_2_O_2_ accumulation during control as well as in salt stress conditions, implying that *OsPLDβ1* plays an important role in oxidative burst (Figure 4C). To add more evidence to the findings, we determined the ROS-scavenging enzymatic activity before and after exposure to salt stress. Antioxidant enzymatic activities of CAT, SOD, and ascorbate peroxidase (APX) were significantly elevated in *Ospldβ1* mutant lines compared to WT plants in response to salt stress (Figures 4D–F). During salt stress, CAT activity decreased markedly in WT plants to 30.80 µmol H_2_O_2_ min^-1^ mg^-1^, whereas the reduction was comparatively lower in *Ospldβ1-1* (48.48 µmol H_2_O_2_ min^-1^ mg^-1^) and *Ospldβ1-2* (47.38 µmol H_2_O_2_ min^-1^ mg^-1^) (Figure 4D). There was a noticeable elevation in the SOD activity of *Ospldβ1* mutant compared to WT, with a maximum level of 2.5 unit/mg reached in the *Ospldβ1-2* line (Figure 4E) during salt stress. Similarly, APX activity was higher in the *Ospldβ*1 mutant lines, and the maximum increase was observed in the *Ospldβ1-2* line, compared to WT in response to salt treatment (Figure 4F). Collectively, these results indicate that the *OsPLDβ1* mutation enhances salt stress tolerance in rice by improving the ROS-scavenging capacity.

### 3.5 Expression of stress-responsive ion transporter genes in *Ospldβ1* lines upon salinity stress

The Na^+^/H^+^ exchanger 1 (NHX1) is the most well-known transporter for vacuolar Na^+^ sequestration. Many organisms, including rice and *Arabidopsis*, exhibit improved salt tolerance and growth rate under overexpression of NHX1 (Apse et al., 1999; Fukuda et al., 2004). NHX1 showed significant differences in expression between WT and *Ospldβ1* mutants under salt stress condition. Expression of the *NHX1* was induced significantly in the *Ospldβ1* mutant (Figure 5A). The *TPC1* (two-pore channel 1) expression increased in the *Ospldβ1* mutant under salt stress compared to WT lines (Figure 5B). When Na^+^ is present in higher concentrations or the cells have a high Na^+^/K^+^ ratio, plant potassium transporters such as the K+ channels (Shaker family) and transporters (HKT and HAK) can either directly or indirectly regulate Na^+^ transport by transporting Na^+^ or buffering cells against Na^+^ uptake by upholding rigorous K+ homeostasis (Platten et al., 2006). The induction of *HKT 2;1* by more than 4-fold increased after 48-h NaCl (150 mM) treatment in *Ospldβ1* mutant leaves, while *HKT 2;1* was not induced in WT leaves (Figure 5C). Furthermore, the other K+ transporter; *HKT 1;1* and HAK1 showed similar upregulation specific for the *Ospldβ1* mutant during salt stress treatment. *HKT 1;1* was induced 4-fold in *Ospldβ1-1* and 2.5-fold in *Ospldβ1*-2, while it was declined in WT lines (Figure 5D). *HAK1* expression increased in both *Ospldβ1-1* and *Ospldβ1*-2 under 48-h salt stress; at the same time, a stronger decrease was observed in WT lines, with nearly undetectable mRNA levels by 48 h of salt stress (Figure 5E). These results indicate that *OsPLDβ1* negatively regulates the expression of ion transporter genes, which could further affect salt stress tolerance levels in rice.

**FIGURE 5:**
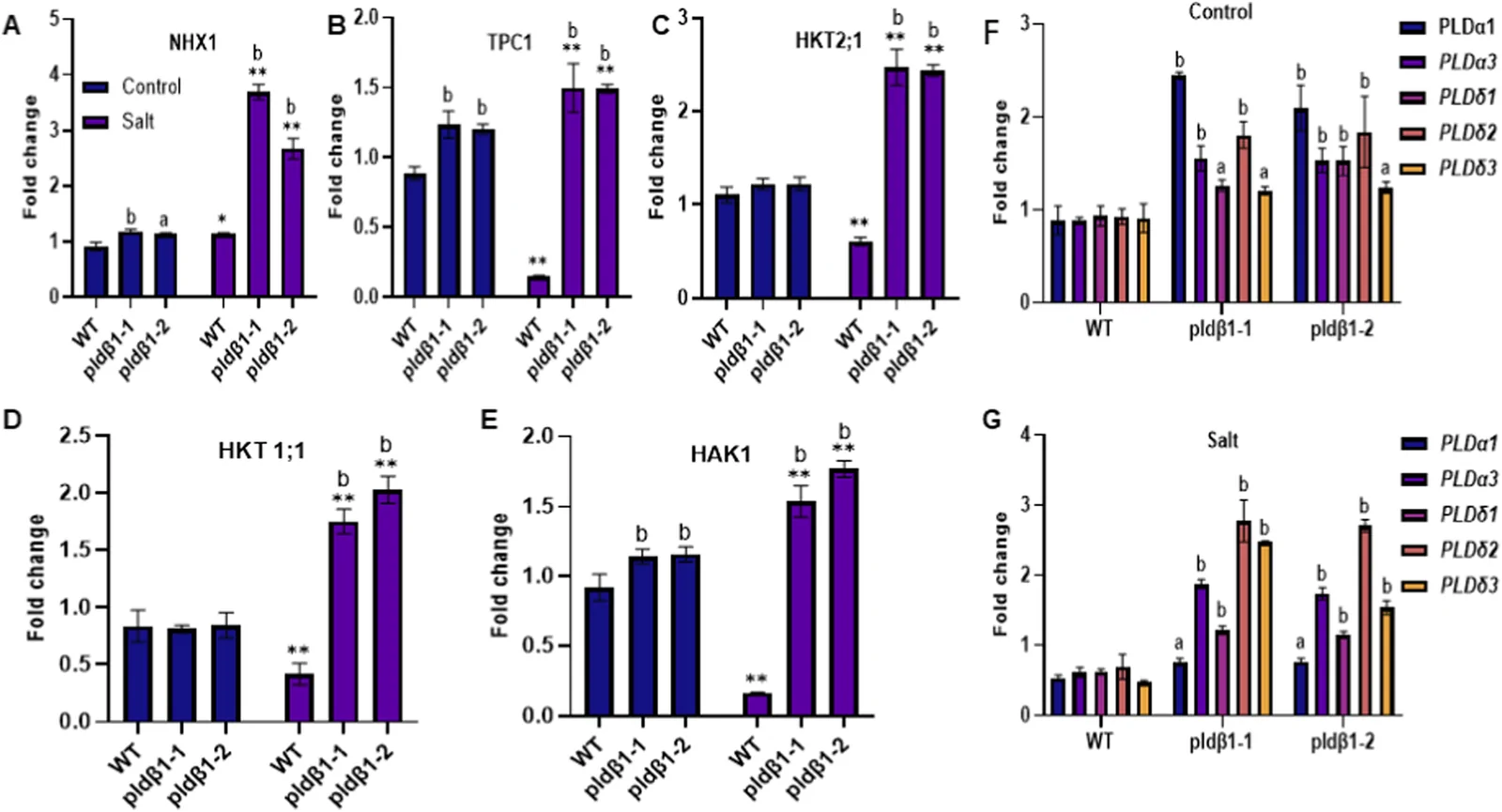
Transcriptional expression analysis of stress-responsive ion transporter genes. The y-axis shows the relative expression level normalized to the WT under normal conditions. Relative expression levels of *NHX1* **(A)**, *TPC1* **(B)**, *HKT2;1* **(C)**, *HKT1;1* **(D)**, and *HAK1* **(E)** in WT, *Ospldβ1-1*, and *Ospldβ1-2* lines under control and salt treatment. qRT-PCR analyses of PLD (*α1, α3, δ1, δ2,* and *δ3*) expression in WT, *Ospldβ1-1*, and *Ospldβ1-2* lines during normal and salt stress conditions. **(F,G)** RNA was extracted from the vegetative state of plant leaves. Values are expressed as means of three biological replicates (three different plants) and three technical replicates ± SD. Letters and symbols indicate a significant difference between means (*p* ≤ 0.05 or 0.01, one-way or two-way ANOVA Tukey HSD *post hoc* test). Different letters indicate mutant vs. WT differences: (a) increase *p* ≤ 0.05, (b) increase *p* ≤ 0.01, (c) decrease *p* ≤ 0.05, and (d) decrease *p* ≤ 0.01. Asterisks indicate differences in control vs. salt stress conditions: *p ≤ 0.05; **p ≤ 0.01.

Based on the previous report, the expression of PLD genes was upregulated under abiotic stress, with these protein products positively regulating the ability of plants to adapt to stress conditions. We further examined whether the expression of the rice PLD members (*α1, α3, δ1, δ2,* and *δ3*) have any changes in *Ospldβ1* mutant lines under salt stress and control conditions. To investigate the role of *OsPLDβ1* in transcriptional regulation PLD isoforms, the *Ospldβ1* mutant plants and the WT were exposed to salt stress, and then qRT-PCR was used to determine the expression levels of PLD members (*α1, α3, δ1, δ2, δ3*). RNA levels of *PLDα1*, *PLDα3*, *PLDδ1*, *PLDδ2*, and *PLDδ3* genes were significantly induced in the plants’ *ospldβ1* mutant lines compared to WT lines during normal growth conditions (Figure 5F). The transcript level of expression level the *PLDα1* gene was slightly higher in *Ospldβ1* mutant plants lines than in the WT after 2 days of salt stress treatment (Figure 5G). At the same time, *PLDδ2* and *PLDδ3* transcript levels markedly increased in the *Ospldβ1* mutant compared to WT during salt stress. The 2-fold induction of both *PLDα3* and *PLDδ1* increased after 48-h NaCl (150 mM) treatment in the *Ospldβ1* mutant compared to WT leaves. These results suggested that *OsPLDβ1* regulates transcription of key stress-responsive PLD isoforms, indicating that knockout of *OsPLDβ1* contributed to increased salt stress in rice.

### 3.6 *OsPLDβ1* mutation mitigates salt stress-induced reductions in agronomic performance and photosynthetic efficiency

Boosting rice productivity under salinity stress is one of the main objectives of salt-tolerance breeding programs. We examined the agronomic performance of *Ospldβ1* mutant plants and WT plants under salinity stress. Three-month-old WT and *Ospldβ1* mutant plants were watered with 0 mM and 100 mM NaCl till completion of seed filling and maturation stages (Figures 6A,B). At the maturation stage, there were no significant differences detected in the main agronomic parameters during control conditions of both *Ospldβ1* mutant and WT (Figure 6C). After 100 mM NaCl treatment for 50 days, 90% of WT plant parts were completely dried; at the same time, 50% of *Ospldβ1* mutant plant parts stayed green after the strong salinity treatment (Figure 6D). Furthermore, we measured chlorophyll content, which is a major indicator for plant damage under stress conditions. We observed that the *Ospldβ1* mutant plants exhibited higher chlorophyll content compared with WT plants under salt stress (Figures 6F, 7A).

**FIGURE 6:**
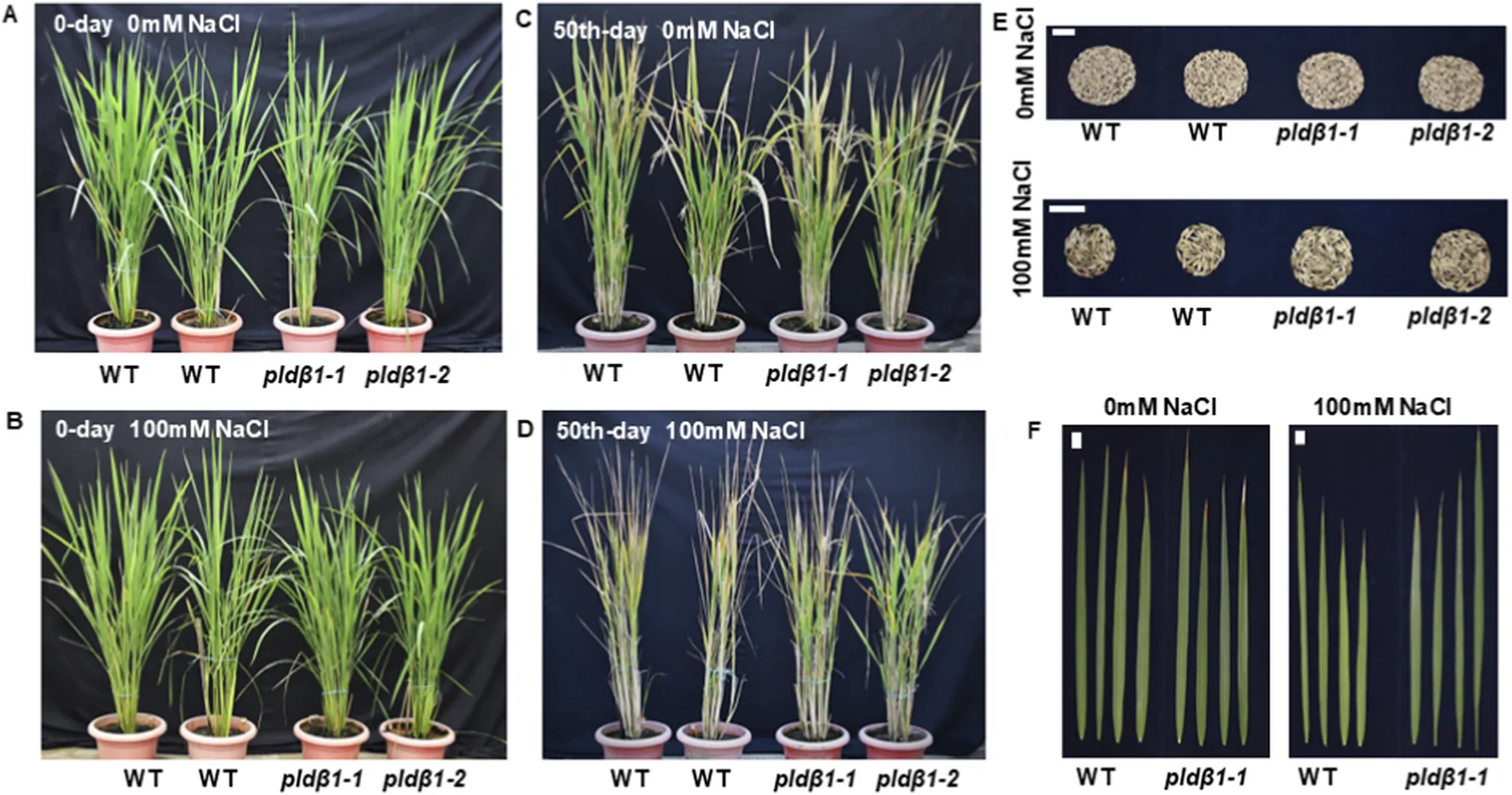
Comparing the plant morphology of mutants at the maturation stage under salt stress. **(A,C)** Plant morphology of WT, *Ospldβ1-1*, and *Ospldβ1-2* (3-month-old) mutants without salt treatment **(B,D)** Plant morphology of WT*, Ospldβ1-1*, and *Ospldβ1-2* mutants during salt treatment. **(E)** Morphology and appearance of bulk grains of WT*, Ospldβ1-1*, and *Ospldβ1-2* mutants. **(F)** Morphology and color changes of the flag leaves during salt treatment. Scale = 2 cm.

**FIGURE 7:**
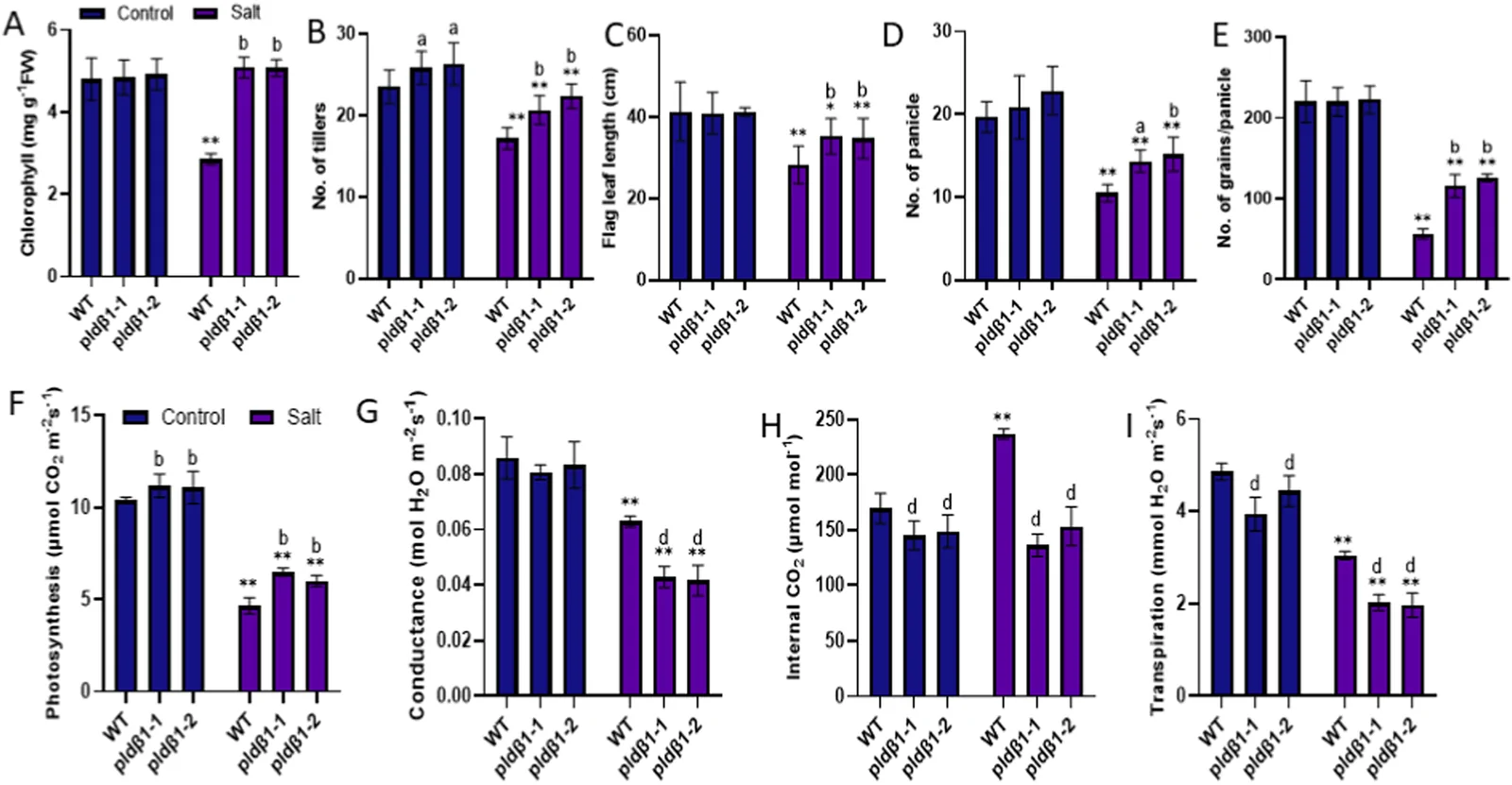
Agronomic performance and photosynthetic parameters under salt stress conditions. **(A)** Chlorophyll level, **(B)** tiller number per plant, **(C)** flag leaf length, and **(D)** Number of panicles per plant and **(E)** Grains per panicle of WT, *Ospldβ1-1*, and *Ospldβ1-2* plants under 0 mM salt and 100 mM salt treatment conditions. **(F)** Leaf photosynthetic rate (*Pn*), **(G)** stomatal conductance (*gs*), **(H)** intercellular CO_2_ concentration (*Ci*), and **(I)** transpiration rate (*Tr*) of WT and mutant rice plants subjected to 0 mM salt and 100 mM salt treatment. Letters and symbols indicate a significant difference between means (*p* ≤ 0.05 or 0.01, one-way or two-way ANOVA; Tukey HSD *post hoc* test). Different letters indicate differences in mutant vs. WT: (a) increase *p* ≤ 0.05, (b) increase *p* ≤ 0.01, (c) decrease *p* ≤ 0.05, and (d) decrease *p* ≤ 0.01. Asterisks indicate differences in control vs. salt stress conditions: *p ≤ 0.05; **p ≤ 0.01.

Under control conditions, indistinguishable differences were examined between WT and *Ospldβ1* mutant plants in the aspects of tiller number, panicle number, and flag leaf length. Conversely, when the plants were exposed to salt stress, the *Ospldβ1* mutant lines maintained improved agronomic performance compared with the WT. The mutant plants exhibited 21 and 23 effective tillers compared with 18 in the WT, representing a 16.7% increase (Figure 7B). Similarly, panicle and flag leaf length in the *Ospldβ1* mutant slightly improved than the WT (Figures 7C,D). Salt stress caused a significant reduction in the total number of grains per panicle in the *Ospldβ1* mutant lines as well as in the WT lines. Nevertheless, the *Ospldβ1* mutant lines retained a higher number of grains per panicle (109 and 115) than the WT (55), representing an approximately 99.2% increase under salt stress (Figures 6E, 7E). All these analyses indicate that mutation of *OsPLDβ1* expression affects agronomic traits in rice under salt stress condition.

Considering the divergence in salt tolerance level between the WT and *Ospldβ1* mutant plants, we further explored the difference in their photosynthetic capacity at the vegetative state (2-month-old plant). We quantified main gas exchange parameters in mutants and WT plants during long-term moderate-salt treatment. The intercellular CO_2_ concentration (Ci), photosynthetic rate (Pn), and transpiration rate sharply decreased under sustained moderate salt stress in the *Ospldβ1* mutant plants as well as in the WT plants (Figures 7F–I). Under control conditions, the *Ospldβ1* mutant lines exhibited a slightly lower stomatal conductance (gs) (0.0759 and 0.0822 mol H_2_O m^-2^ s^-1^ in *Ospldβ1-1* and *Ospldβ1-2*, respectively) than the WT (0.0831 mol H_2_O m^-2^ s^-1^) (Figure 7G). Similarly, the intercellular CO_2_ concentration (Ci) was lower in the *Ospldβ1* mutant lines (133.2 and 148.9 μmol mol^-1^) than in the WT (164.1 μmol mol^-1^), accompanied by lower transpiration rates (4.18 and 4.44 mmol H_2_O m^-2^ s^-1^ in the *Ospldβ1* mutant lines compared with 4.81 mmol H_2_O m^-2^ s^-1^ in the WT) (Figures 7H,I). Even under these conditions, the net photosynthetic rate (Pn) was slightly higher in the mutant lines (11.06 and 11.09 µmol CO_2_ m^-2^ s^-1^) than in the WT (10.36 µmol CO_2_ m^-2^ s^-1^). Under salinity stress, Pn decreased in all genotypes but remained significantly higher in the *Ospldβ1* mutant lines (6.33 and 5.75 µmol CO_2_ m^-2^ s^-1^) than in the WT (4.89 µmol CO_2_ m^-2^ s^-1^) (Figure 7F). This higher photosynthetic performance in the *Ospldβ1* mutant lines occurred despite the lower stomatal conductance (0.0452 and 0.0443 mol H_2_O m^-2^ s^-1^ in *Ospldβ1-1* and *Ospldβ1-2*, respectively, versus 0.0558 mol H_2_O m^-2^ s^-1^ in the WT) (Figure 7G), lower intercellular CO_2_ concentration (148.4 and 166.8 μmol mol^-1^ versus 227.6 μmol mol^-1^ in the WT) (Figure 7H), and lower transpiration rates (2.13 and 2.10 mmol H_2_O m^-2^ s^-1^ versus 2.69 mmol H_2_O m^-2^ s^-1^ in the WT) (Figure 7I). These results indicate that the *Ospldβ1* mutant lines maintained higher photosynthetic performance under salinity stress despite the reduced stomatal conductance and transpiration. Furthermore, the transpiration rate was markedly lower in the *Ospldβ1* mutants than in the WT, suggesting a divergence in how the two genotypes manage gas exchange under salinity. These results suggest that *Ospldβ1* mutant rice exhibited better photosynthetic capacity in a long-term salinity treatment.

## 4 Discussion

Rice (*O. sativa* L.) serves as a fundamental food source for approximately 50% of the world’s population; yet its cultivation faces increasing hazards due to soil salinity (Shrestha et al., 2020; Kim et al., 2020). Salt stress disrupts ion homeostasis, induces oxidative and osmotic stress, and impairs photosynthetic efficiency, thereby decreasing yield (Hussain et al., 2022). Due to climate change and unsustainable irrigation methods increasing soil salinity, improving salt tolerance in rice has emerged as a primary target in agricultural research (Paul and Bhatia, 2025). PLD enzymes, especially *OsPLDβ1*, are essential for membrane lipid signaling and stress adaption (González-Mendoza et al., 2021). The exact function of *OsPLDβ1* in regulating responses to salt stress is still uncertain (Zhong et al., 2024). The present study examines the physiological and molecular effects of *OsPLDβ1* knockout, positing that its disruption improves rice salt tolerance. These findings demonstrate that *OsPLDβ1* knockout enhances salt tolerance by improving ROS enzymatic homeostasis and upregulating the expressions of stress-responsive ion transporter genes. These findings identify *OsPLDβ1* as a viable target for genetic alteration, presenting a prospective strategy for the development of salt-tolerant rice cultivars.

### 4.1 *Ospldβ1* mutant growth and development under salt stress

The knockout of *OsPLDβ1* significantly improved rice’s ability to survive salt stress, as evidenced by better seed germination, chlorophyll retention, and shoot and root development (Figure 1). These findings underline the crucial regulatory role of *OsPLDβ1* in the morphological and physiological responses of rice to salinity (Cao et al., 2021). One notable observation was that the *Ospldβ1* mutants demonstrated considerably greater chlorophyll retention relative to WT plants during salt stress (Figures 1B,C). Chlorophyll degradation is a common result of salinity, leading to impaired photosynthesis and growth (Tanaka and Ito, 2024). The maintenance of chlorophyll content in the mutant plants shows that the loss of *OsPLDβ1* helps attenuate salt-induced chlorophyll degradation, promoting greater photosynthetic efficiency and plant development, which is critical for salt tolerance (Sun et al., 2021; Yan et al., 2022).

The study also discovered that *Ospldβ1* mutant seedlings had a better germination rate and growth compared to WT seeds under salt stress (Figures 1G–I). This suggests an increased ability to withstand osmotic stress and ionic toxicity (Thorat et al., 2018). The enhanced germination rate aligns well with the findings of prior research on ion transporters including NHX1 and HKT1, which help in osmotic adjustment and seedling establishment in saline conditions (Rao et al., 2021). Moreover, the higher root and shoot growth in the mutants further supports this as a stronger root system facilitates better water and nutrient uptake, important for survival under osmotic stress. These results coincide with findings from other research where genetic modifications in root architecture contributed to enhanced salt tolerance (Julkowska et al., 2017).

### 4.2 Salt tolerance of mutants at the vegetative stage and physiological–biochemical responses contributing to stress resilience

The hydroponic experiment results indicated that *Ospldβ1* mutant rice plants exhibit improved tolerance to salt stress during the vegetative stage compared to WT plants (Figures 2D, 3A). Interestingly, the two independent *Ospldβ1* mutant lines exhibited different survival rates during the 2-week-old vegetative stage salt stress assay. The *Ospldβ1-2* shows higher survival than *Ospldβ1-1*. However, the difference in survival is unlikely to result from differential *OsPLDβ1* expression. The qRT-PCR analysis performed in our previous study (Karippadakam et al., 2026) confirmed that *OsPLDβ1* transcript levels were undetectable in both mutant lines, indicating successful disruption of the target gene. Notably, both mutant lines exhibited comparable salt tolerance during the 2-month-old vegetative stage and at the reproductive stage, with no marked differences in their physiological or agronomic performance. Based on the observations, we suggest that the variation in survival may be specific to the early vegetative-stage assay, but further studies are required to determine the factors underlying this stage-specific variation. Importantly, both mutants consistently exhibited significantly improved salt tolerance than the WT plants, supporting the conclusion that loss of *OsPLDβ1* enhances salt stress tolerance. When exposed to NaCl in a nutrient medium, mutant lines retained superior growth characteristics, suggesting an improved capacity to cope with salinity-induced stress. These findings correlate with those of earlier research suggesting that genetic modifications targeting stress-responsive pathways can greatly boost salt tolerance in rice (Singh et al., 2021; Luo et al., 2023). The enhanced salt tolerance found in *Ospldβ1* mutants is likely attributable to several physiological changes, particularly in root architecture, osmotic balance, and oxidative stress regulation (Khan et al., 2018a). Under saline conditions, the mutant plants (14 days old) demonstrated greater root number and length, which may permit improved water and nutrient uptake despite osmotic stress (Figures 3F,H). Moreover, the improved root development, characterized by larger root length and density, facilitates more efficient water and nutrient uptake, which is crucial for maintaining cellular homeostasis under saline conditions. This root development is essential for preventing sodium toxicity by enabling greater absorption of necessary ions (Rubio et al., 2020). Chlorophyll degradation is a usual consequence of salt stress, but the mutants’ improved retention indicates superior protection against photoinhibition. This contributes to sustained biomass production, especially in saline environments (Sadiq et al., 2019). The increased chlorophyll content (Figure 7A; Supplementary Figure S3) in mutant lines suggests enhanced photosynthetic efficiency, a vital component for sustaining growth under stress conditions.

Proline works as an osmoprotectant, stabilizing proteins and cellular structures under stress (Siddique et al., 2018). The higher proline accumulation in the mutants indicates improved osmotic balance regulation, facilitating salt stress adaption. One possible explanation for the enhanced survival of *Ospldβ1* mutant plants under salt stress is the fact that they upregulate osmoprotectant molecules, such as proline (Zulfiqar and Ashraf, 2023). Proline accumulation has been well documented as a crucial process for osmotic adjustment, safeguarding cellular structures and stabilizing proteins under stress conditions (Forlani et al., 2019). The observed increase in proline levels in mutant lines further supports this theory. Additionally, the reduction in MDA level and electrolyte leakage in mutant plants shows reduced membrane lipid peroxidation, signaling higher membrane integrity during salt stress (Chauhan and Sanadhya, 2019). Furthermore, the reduced MDA and electrolyte leakage levels in the mutants provide insights into greater membrane integrity as oxidative damage can be prevented (Figures 3B,C). This shows that the loss of *OsPLDβ1* may contribute to enhanced cellular integrity by decreasing oxidative stress-induced damage.

### 4.3 Role of *OsPLDβ1* in ROS regulation

ROS perform a dual role in plants, operating both as signaling molecules and as agents of oxidative damage, particularly under stress conditions (Azarabadi et al., 2016). The results of this study reveal that *OsPLDβ1* negatively regulates the ROS scavenging mechanism under salt stress. *OsPLDβ1* knockout in rice leads to improved antioxidant enzymatic activity (Figures 4D–F), enhancing cellular protection and survival under saline conditions. These findings underline the important role of *OsPLDβ1* in modulating the oxidative stress response of plants generated by exposure to salt stress.

In response to salt stress, the *Ospldβ1* mutant plants demonstrated considerably increased activity of vital antioxidant enzymes compared to WT plants (Figures 4D–F). Notably, the activities of APX, SOD, and CAT were increased, indicating an improved ROS detoxification mechanism (Mittler, 2017; Pandey et al., 2017). These enzymes are crucial for neutralizing ROS and limiting oxidative damage to cellular components such as lipids, proteins, and nucleic acids. The enhanced activity of these enzymes in *Ospldβ1* mutants shows that the knockout boosts the ROS scavenging ability, hence alleviating oxidative stress and enhancing stress tolerance (Figures 4A–C).

The improved ROS scavenging in the mutant plants appears to be mediated by the overexpression of antioxidant enzymes. SOD transforms superoxide radicals into H_2_O_2_, which is subsequently broken down by CAT and APX into water and oxygen, successfully preventing cellular damage (Smirnoff and Arnaud, 2019). Along with enzymatic antioxidants, the increase of non-enzymatic antioxidants including proline and ascorbic acid in the mutant plants further supports the improved detoxification of ROS (Renzetti et al., 2024). The higher amounts of proline seen in the *Ospldβ1* knockout lines imply that proline also contributes to cellular defense by stabilizing proteins and membranes, limiting ROS-induced damage.

Previous reports state that the PLD can function as both a positive and negative regulator of ROS generation, depending on the specific isoform and stress (Zhang et al., 2009; Yamaguchi et al., 2019). The H_2_O_2_ accumulation in the *OsPLDβ1* knockout lines reduced along with increased APX activity, suggesting enhanced antioxidant capacity and more efficient detoxification of H_2_O_2_ under salt stress. In *Arabidopsis*, *AtPLDα1* and PA directly regulate the activity of the NADPH oxidase, thereby promoting ROS production required for ABA-mediated stomatal closure (Zhang et al., 2009). In rice, *OsPLDβ2* functions as a negative regulator of the catalase gene *OsCATA*, and suppression of *OsPLDβ2* enhances *OsCATA* expression, resulting in increased H_2_O_2_ scavenging and reduced oxidative damage (Yamaguchi et al., 2019). These observations suggest that *OsPLDβ1* positively influences ROS homeostasis and that its disruption enhances the antioxidant defense system, thereby promoting more efficient H_2_O_2_ detoxification. However, it is not possible to determine whether the reduced H_2_O_2_ accumulation primarily results from decreased ROS production, enhanced antioxidant scavenging, or a combination of both mechanisms. Nevertheless, our findings, together with those of previous studies on other PLD isoforms, support the concept that PLDs are important regulators of ROS homeostasis. Additionally, the downstream regulatory mechanisms appear to be isoform-specific and dependent on the type of abiotic stress.

The increased ROS detoxification in *Ospldβ1* mutants is directly linked with reduced oxidative damage, as indicated by lower MDA levels and decreased electrolyte leakage, implying less lipid peroxidation and membrane damage. Additionally, PI staining showed fewer dead cells, and CoroNa Green staining showed lower Na^+^-associated fluorescence in the roots of *Ospldβ1* mutants compared with WT under salt stress (Supplementary Figure S5). Furthermore, the *Ospldβ1* knockout lines showed better chlorophyll retention as well as improved growth parameters under saline conditions, indicating that the enhanced ROS scavenging capability preserved vital cellular functions, including photosynthesis and energy metabolism, thereby enhancing overall plant vigor (Zhang Z. et al., 2019). The results of this study reveal that *OsPLDβ1* is an essential regulator of ROS homeostasis in rice during salt stress. By implementing gene-editing tools such as CRISPR–Cas9 to target *OsPLDβ1*, it may be possible to generate rice varieties with increased salt tolerance, presenting a viable strategy for sustainable rice production in saline-affected regions.

### 4.4 Transcriptional regulation of stress-responsive genes in *OsPLDβ1* knockout plants

In this study, *OsPLDβ1* knockout drastically affected the transcriptional regulation of essential stress-responsive genes involved in ion homeostasis, osmotic regulation, and signal transduction, revealing the significance of *OsPLDβ1* as a negative regulator of salt stress adaption. The elevated levels of salt-responsive genes (*TPC1, NHX1, HAK1, HKT1;1, and HKT2;1*) in the *Ospldβ1* mutant plants (Figures 5A–E) may lead to activation of ion transport pathways associated with ion homeostasis under salt stress (Roychoudhury et al., 2021). The activation of these genes presumably facilitates K^+^ retention and Na^+^ exclusion, enabling the mutant plants to keep their ionic balance and avoid salt-induced toxicity (Ragel et al., 2019).

A notable observation in this study was the substantial *NHX1* upregulation, which encodes a vacuolar Na^+^/H^+^ antiporter. This protein is needed for sequestering excess sodium ions into vacuoles, thus lowering cytoplasmic sodium toxicity (Khan et al., 2018b). Enhanced *NHX1* expression has been connected with improved salt tolerance in rice and other plant species, and the findings of this study show that *OsPLDβ1* knockout indirectly increases *NHX1*-mediated sodium compartmentalization (Figure 5A). This correlates with earlier findings indicating that *NHX1* overexpression promotes salt tolerance by maintaining osmotic balance and shielding cells from ionic stress (Zhao et al., 2019).

In addition to *NHX1*, the enhanced expression of *TPC1*, a two-pore calcium channel implicated in calcium-dependent stress signaling, shows the importance of calcium signaling in salinity stress resilience. TPC1 is involved in the regulation of ion fluxes and Ca^2+^-mediated signaling during the abiotic stresses, including salinity (Liu et al., 2018). Suggesting that *OsPLDβ1* knockout may improve calcium signaling to bolster stress adaptation (Figure 5B). This discovery is consistent with studies indicating that higher TPC1 activity leads to greater salt stress tolerance by regulating ion fluxes and activating protective pathways (Feijóo-Bandín et al., 2017).

The increased expression of *HAK1*, a high-affinity potassium transporter, *HKT1;1*, and *HKT2;1* further supports the concept that *OsPLDβ*1 adversely affects ion transport mechanisms under saline conditions (Figures 5C,D). *HAK1* is necessary for potassium absorption, helping maintain a high K^+^/Na^+^ ratio, a key predictor of salt tolerance (Chen et al., 2015). The enhanced expression of *HAK1* in *Ospldβ1* knockout mutants suggests greater potassium retention, which lessens the impact of sodium toxicity (Figure 5E). Previous studies have shown that *OsHKT2;1* mediates Na^+^ uptake under potassium-deficient conditions, whereas HKT1 family transporters regulate Na^+^ retrieval and redistribution to prevent excessive accumulation in sensitive tissues. The salt tolerance is determined by the coordinated activity of multiple Na^+^ transporters rather than by a single HKT transporter. In the present study, the simultaneous upregulation of *OsHKT1;1* and *OsHKT2;1*, together with reduced Na^+^ accumulation in *Ospldβ1* mutant plants, indicates that knockout of *OsPLDβ1* may promote a more efficient Na^+^ transporter mechanism, such as Na^+^ uptake, redistribution, and shoot Na^+^ exclusion in rice, thereby minimizing ionic toxicity and enhancing salt tolerance (Zhang et al., 2018; Jabnoune et al., 2009; Miyamoto et al., 2015, Wang et al., 2015). Previous studies have shown that *OsHKT2;1*-mediated Na^+^ influx can be suppressed despite substantial transcript accumulation. Consequently, increased transcript levels do not necessarily correspond to increased transporter activity (Horie et. al., 2007). Therefore, the increased *OsHKT2;1* expression observed in the leaves of mutant plants reflects an enhanced capacity for Na^+^ transport; however, the simultaneous activation of other Na^+^ transport systems may prevent excessive Na^+^ accumulation in plant tissues through a coordinated regulation of multiple ion transporter.

*OsPLDβ1* has an significant role in transcriptional regulation of other rice PLD isoforms such as *PLDα1, PLDα3, PLDδ1, PLDδ2,* and *PLDδ3* genes. Those genes were markedly induced in the *Ospldβ1* mutant lines in comparison with WT lines during normal conditions (Figure 5F). Furthermore, the transcript levels of *PLDα1, PLDα3, PLDδ1, PLDδ2,* and *PLDδ3* are slightly higher in *Ospldβ1* mutant plants lines than in the WT after 2 days of salt stress treatment (Figure 5G). The *pldα1* and *pldδ* mutants showed increased sensitivity to salt stress in a plate assay. Dehydration activates *PLDα1* and *PLDδ,* and the mutants exhibit increased sensitivity to hyperosmotic stress, showing severely reduced growth (Bargmann et al., 2008). The increased basal expression of *OsPLDα3* observed in the *Ospldβ1* mutant lines is a significant observation as *PLDα3* has previously been reported to positively regulate plant responses to salinity and water deficit through modulation of PA production (Hong et al., 2008). These results align well with the RT-PCR results of *Ospldβ1* mutant plants during salt stress.

Additionally, the PLD isoforms were transcriptionally upregulated in the *Ospldβ1* mutant lines even under control conditions, suggesting a compensatory response to the loss of *OsPLDβ1* gene function. Similar compensatory regulation among PLD family members has been reported previously. Rapid PA accumulation during wounding stress was observed even in *PLDα*-deficient plants, indicating that other PLD isoforms can compensate for the loss of *PLDα* and PA production (Zien et al., 2001). Likewise, *PLDδ* expression was significantly upregulated in anti-*PLDα* plants under control conditions, further supporting a possible compensatory response among PLD isoforms (Mane et al., 2007). In addition, Hong et al. (2008) demonstrate that *PLDα3* is active under *PLDα1* assay conditions but not under *PLDβ*-specific conditions, suggesting that PLD isoforms possess distinct functional properties. Although these findings support the possibility of compensatory regulation among PLD family members, PA accumulation and PLD enzymatic activities were not determined in the present study. Therefore, it remains unclear whether the enhanced salt tolerance of the *Ospldβ1* mutant lines is associated with altered basal PA signaling or other downstream regulatory mechanisms. Further studies on enzymatic analyses will be required to clarify the compensatory response. Another possibility for the upregulation of PLD isoforms in the mutants is genetic compensation through transcriptional adaptation triggered by mutant mRNA degradation. The absence of *OsPLDβ1* transcripts in the knockout lines suggests that the CRISPR-/Cas9-induced frameshift mutations and premature termination codons may result in degradation of the transcripts through nonsense-mediated mRNA decay (NMD). mRNA degradation has been reported to trigger transcriptional adaptation, which can lead to the upregulation of related genes to compensate for the loss of a particular gene (El-Brolosy et al., 2019). The increased upregulation of other *OsPLD* family members in association with the loss of *OsPLDβ1* transcripts is consistent with a potential transcriptional adaptation response and may contribute to the enhanced salt tolerance of the *Ospldβ1* mutants. Previous studies have also demonstrated functional redundancy among PLD family members. In *Arabidopsis*, knockout of individual PLD genes did not significantly affect the hypersensitive response (HR), suggesting substantial functional redundancy among PLD isoforms. In contrast, loss of one of the major PLD isoforms (*PLD*δ) was sufficient to significantly affect defense responses (Johansson et al., 2014). Thus, the increased expression of other *OsPLD* isoforms may compensate for the loss of *OsPLDβ1* and could contribute to the enhanced salt tolerance. However, whether these transcriptional changes in PLD isoforms directly compensate for *OsPLDβ1* function remains to be experimentally determined.

PA is an important signaling molecule in plant stress responses; future studies should determine how *OsPLDβ1* knockout influences PA production and downstream signaling under salt stress. The transcriptional variations found in this study imply that *OsPLDβ1* plays a vital role in salt stress signaling, presumably through interactions with hormones such as abscisic acid (ABA) and ROS, which are known for regulating stress responses. The increase in the expressions of important genes involved in osmotic regulation, ion transport, and cellular protection demonstrates that *OsPLDβ1* works as a transcriptional repressor, influencing the activation of stress-adaptive mechanisms (Kesawat et al., 2023).

### 4.5 Impact on agronomic performance and photosynthesis efficiency

The results of this study reveal that the *OsPLDβ1* knockout positively affected important agronomic traits under salt stress, resulting in improved photosynthetic efficiency and better maintenance of grain production per plant. Despite the deleterious impact of salinity on both WT and mutant lines, the *Ospldβ1* mutant plants displayed substantially greater grain production, increased tiller number, longer flag leaves, and improved panicle formation (Figures 7B–E). These findings emphasize the possibility of *OsPLDβ1* gene editing as a technique to boost salt tolerance without considerably compromising production (Zahra et al., 2022; Parida et al., 2022; Amokrane et al., 2024).

The ability of *Ospldβ1* mutants to sustain superior growth and reproductive performance under saline conditions has a strong connection to their enhanced physiological and biochemical responses. The higher tiller number and longer flag leaves suggest superior resource allocation and photosynthetic efficiency, which are crucial determinants in determining overall plant biomass and grain production (Ding et al., 2023). Panicle development, a major determinant of grain production in rice, was better maintained in the mutant lines than in the WT under salt stress conditions. These results correspond with those of previous studies demonstrating that plants with improved osmotic adjustment and ion homeostasis showed better reproductive success even under harsh environmental conditions (Martínez-Ballesta et al., 2020).

Another important aspect of this study is the improved photochemical efficiency seen in *Ospldβ1* mutant plants. Photosynthetic performance is a key indicator of plant productivity, and salt stress often leads to photoinhibition and lower electron transport efficiency in plants (Muhammad et al., 2021). However, chlorophyll retention, higher photosynthesis rate, and lower transpiration activity in mutant lines imply that the *OsPLDβ1* knockout may contribute to better photosynthetic stability under salt stress conditions (Figures 7F–I). The improved photosynthetic performance of the *Ospldβ1* mutant lines may also be associated with enhanced protection against oxidative stress. The mutant lines accumulated lower levels of H_2_O_2_ together with increased APX activity, indicating a more efficient antioxidant defense system. This could be attributed to reduce oxidative damage, lower membrane lipid peroxidation, and more effective ROS scavenging systems in *Ospldβ1* mutant lines. The ability to retain higher photosystem II (PSII) efficiency under stress suggests that these plants have considerable potential for prolonged carbon fixation and biomass accumulation, ultimately translating to increased grain yield (Liu et al., 2019).

However, the reduction in Pn was less severe in the mutant lines, which maintained significantly higher photosynthetic rates than the WT under salt stress condition. These findings suggest that disruption of *OsPLDβ1* mitigates the inhibitory effects of salinity on photosynthesis. Although reduced Gs generally restricts CO_2_ diffusion into the leaf, the decrease in Ci together with the higher Pn suggests that CO_2_ was assimilated more efficiently in the mutant lines rather than accumulating within the intercellular spaces. This result indicates that carbon assimilation was less severely affected in the mutant lines under salt stress despite partial stomatal closure. Previous studies have shown that photosynthesis under salinity is regulated by both stomatal and non-stomatal limitations, including changes in mesophyll conductance and the biochemical capacity for CO_2_ fixation (Flexas and Medrano 2002; Chaves et al., 2009).

Although the *Ospldβ1* mutants showed improved photosynthetic performance, chlorophyll fluorescence parameters (Fv/Fm or ΔF/Fm’) were not measured in the present study. The shift from stomatal to non-stomatal resilience explains why the reduction in Pn was significantly less severe in the mutant compared to the WT. Future measurements of chlorophyll fluorescence (Fv/Fm) may confirm whether the maximum quantum yield of PSII is better maintained in the *Ospldβ1* mutant lines under salt stress and provide a more comprehensive understanding of the underlying physiological mechanisms.

The present study demonstrates that disruption of *OsPLDβ1* enhances salt tolerance through improved antioxidant defense, photosynthetic performance, and regulation of salt-responsive genes; however, the underlying molecular mechanisms still remain to be fully understood. The quantification of PA level and PLD activity was not performed in this current study. Future investigations should, therefore, focus on quantifying PA and PLD activity in *Ospldβ1* mutant and WT plants under both control and salt stress conditions to validate the transcript level of PLD isoforms in the present study and to establish the relationship between *OsPLDβ1* disruption and PA-mediated signaling.

The implications of these results for rice breeding programs and agricultural productivity are enormous. Salinity is a key barrier in rice production, particularly in coastal and irrigated areas where soil salinization is a growing concern. The *OsPLDβ1* knockout technique offers a viable approach to create salt-tolerant rice cultivars with minimal trade-offs in yield (Prabhu et al., 2023). The ability of mutant plants to sustain better growth, reproductive success, and photochemical efficiency under salinity stress indicates that targeting *OsPLDβ1* through CRISPR/Cas9 gene-editing technologies could be a promising breeding strategy for developing stress-resilient rice varieties (Singh et al., 2025; Kanth et al., 2025). Moreover, incorporating *OsPLDβ1* knockout lines into breeding programs could help achieve sustainable rice production in salinity-prone areas, lowering the need on excessive irrigation and soil amendments (Chapagain et al., 2021). Future studies should investigate the long-term agronomic stability of *Ospldβ1* mutant lines under field conditions, as well as their performance in combination with other stress-tolerance traits.

## 5 Conclusion

This study demonstrates that disruption of *OsPLDβ1* is associated with enhanced salt tolerance in rice and provides physiological, biochemical, and transcriptional evidence supporting its involvement in salinity responses. The *OsPLDβ1* knockout lines exhibited enhanced salt tolerance, accompanied by enhanced antioxidant capacity, improved photosynthetic efficiency, and altered expression of ion transport and homeostasis genes. These findings suggest that *OsPLDβ1* may play an important role in the responses of rice cultivars to salinity and represents a promising candidate for further functional studies and crop improvement. The application of *OsPLDβ1* gene editing by CRISPR/Cas9 or other modern biotechnological technologies could be important in producing salt-tolerant rice cultivars. This has major implications for enhancing rice productivity in saline-affected areas, contributing to global food security. Future research needs to concentrate on large-scale field trials to validate these results under natural conditions. Additionally, further molecular research is required to clarify the downstream signaling pathways impacted by *OsPLDβ1*. Understanding these mechanisms will provide deeper insights into plant stress response and help the development of more resilient rice varieties.

## Data Availability

The original contributions presented in the study are included in the article/Supplementary Material, further inquiries can be directed to the corresponding author. Data will be made available on request.
