# Supplementary material for: CRISPR/Cas9-mediated disruption of OsPLDβ1 enhances salt stress tolerance in rice through modulation of antioxidant defense
Source: Front Genome Ed. 2026 Sep 21;8:1904040. doi: 10.3389/fgeed.2026.1904040 (PMC13634860; doi:10.3389/fgeed.2026.1904040)
Supplement: Supplementary file 1 [file DataSheet1.pdf]

Supplementary Files

CRISPR/Cas9 Mediated Disruption of *OsPLDβ1* Enhances Salt Stress Tolerance in Rice Through Modulation of Antioxidant Defense

Sangeetha Karippadakam [a,b], V. Mohan M. Achary [a*], Dalia Vishnudasan [b], Hemangini Parmar[a], Alexander Balamurugan[c], Mallesham Bulle[d], Ganesan Prakash [c], Malireddy K. Reddy[a]

[a] Crop Improvement Group, International Centre for Genetic Engineering and Biotechnology, Aruna Asaf Ali Marg, New Delhi 110067, Delhi, India-110067

[b] School of Biotechnology, Amrita Vishwa Vidyapeetham, Kollam, Kerala 690525, India.

[c] ICAR- Indian Agricultural Research Institute (IARI), Plant Pathology, New Delhi, India, 110012

[d]School of Plant, Environmental and soil Science, Louisiana State University Agricultural Center, Baton Rouge, LA, 70803, USA.

Classification: Personal

Mutants | Chromatogram and sequence confirmation

OsPLDβ1-WT

Ospldβ1-1

Ospldβ1-2

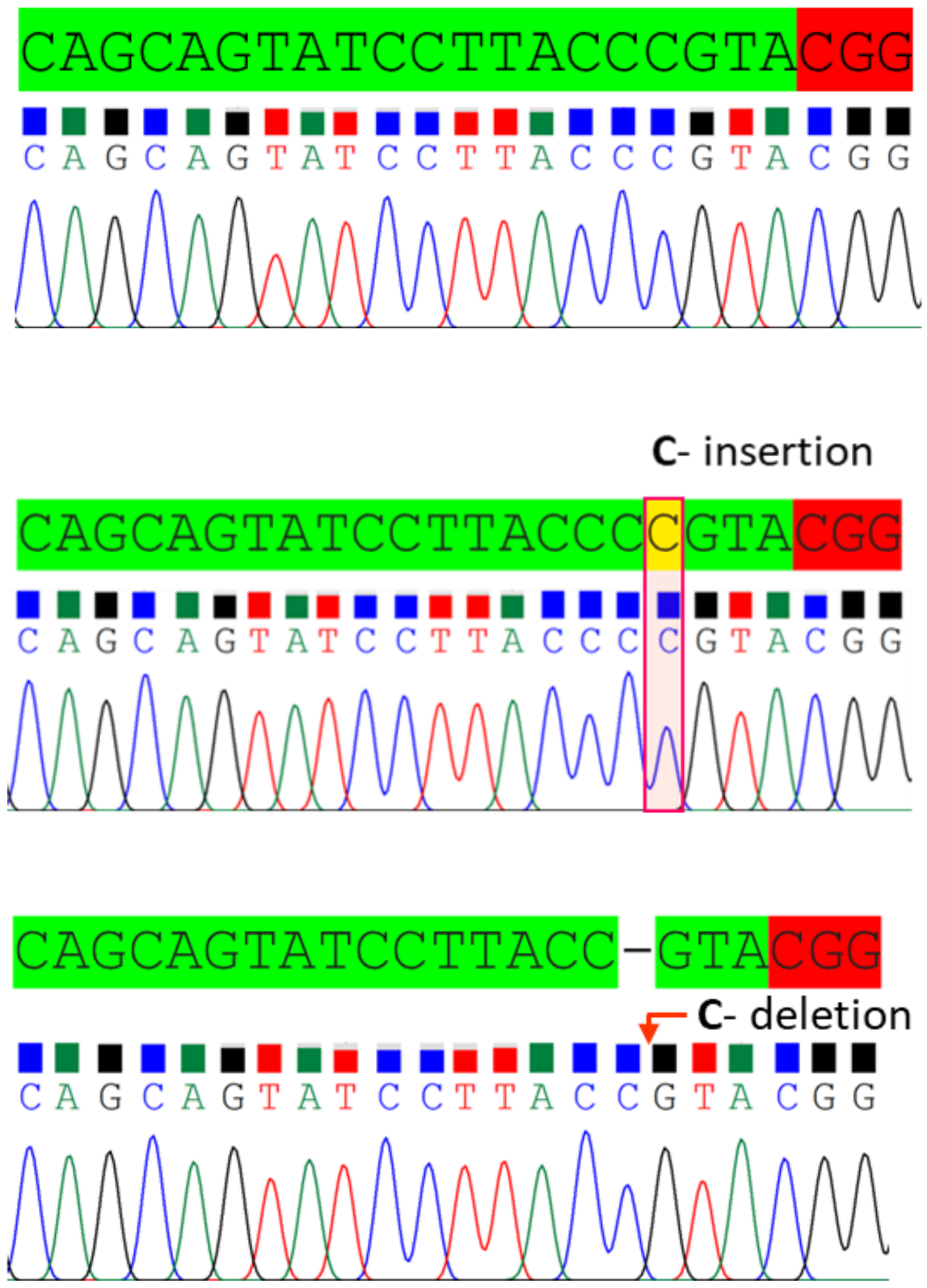

Supplementary Figure 1: Chromatogram and sequence confirmation of CRISPR-Cas9 edited rice lines (*pld β1-1, pld β1-2*) (Karippadakam et al., 2026).

ATGGAGGGCGGAAACCACGGTGGGGGAGGGTACCCCTACCCGCCGCCGCAGCAGTATCCTTACCCcGTACGGGCAGTACCCATACCAGTATCCTCCACC
M E G G N H G G G G Y P Y P P P Q Q Y P Y P V R A V P I P V S S T

GCAGCAGCAGCCGCCGCCGCCCTCCGCCTACCTCTCGCCGTCCCGTTCGTTCCACGGTTACCCCTCCGCGCCGCCGCCGCAGCCGCAGCCGCAGCCGTA
A A A A A A A L R L P L A V P F V P R L P L R A A A A A A A A A V

CGCGCACCATTCCGCCCCGCTCCAGCCTTACCCGCCGCCGCCGCAGCACCATGCCTACCCGCCGCCGCAGCCGCATCCGCCCTCGCCGTACGTCTACGA
R A P F R P A P A L P A A A A A P C L P A A A A A S A L A V R L R

CCCTTACCACGCGCCCGCCGCGGCGTACCCTTCCTACCCGAGCCCCAACCCCAGCCCGTCCATCTCCCCGAGCTCCAGCTTCCACCACCACCCTGAACC
P L P R A R R G V P F L P E P Q P Q P V H L P E L Q L P P P P *

GCCATCGCCATCGCCCTCCGCGCCGTCGTACCCCTCCATTGCGGATGGGCTCGCCAACATGCACGTCTCCGACCGCCACGACTACCCGCCGCCGCCGTC
GCCGGCGGCCGTGCCCGCGGCGTCGTCCCCCTCCGTGCTCCCGCCTTCCGCGTCGTTCCCCGGCGGCGGGAGCAGCCACGGCGGTGGCGGGATGCAGAT
GGTGCCGTACGGCCCTCCTGCCGGAGGGTCGCAGCACGGTGGGATGCAGATGGTGGCGTACGGCTCGCCGGCCGGAGGGTCGCAGCACGGCAGCGTGAG
GCCATCCCTGAAGGTGGTGCTGCTCCACGGCACCCTCGACGTCTGGGTGTACGACGCTCGGAACCTCCCGAACAAGGATCTCTTCTCCAAGAGGGTCGG
CGACCTCCTCGGTCCGCGCCTCATCGGCGCCGTCGGCAGCAAAATGTCCAGCGCCAACATGACCAGCGACCCCTATGTTACCATCCAGGTCTCCTATGC
CACCGTCGCGCGCACCTACGTCGTTCCCAACAATGAGAACCCCGTTTGGACGCAGAATTTCCTTGTCCCTGTTGGGCATGATGCCGCCGAGGTCGAGTT
CGTTGTCAAGGACAATGATGTCTTCGGTGCTCAGCTCATAGGCACAGTGTCGATTCCTGCTGAGAAGCTCCTTTTTGGAGAAAGGATTAACGGCATCTA
CGATGTGCTTGAGTCCAATGGGAAGCCATGCGCTCAAGGTGCTGTGTTGCGGCTGTCCATACAGTACATCCCGGTGGCTCAGCTCAAAATGTATCACCA
TGGTGTCATTGCCGGCCCAGATAGCCTGGGAGTTCCTAATACTTACTTCCCGATGCGGCGTGGCAATAGAGTGACACTGTATCAGGACGCACATGTGCC
TGATGGTTGTCTCCCGGATTTCTGTCTTGATCACGGAATGCGCTATCAGCACGGGCAGTGTTGGCGTGATATCTACGATGCTATATGCCAAGCACGGCG
GTTGATTTACATTGTGGGATGGTCAGTGTTCCACACAATCCACCTCATCAGGGAAGGAGTTGAGAAGATGCCATCACTTGGGGAACTGTTGAAGATGAA
GTCACAGGAAGGGGTGAGGGTGTTGCTTCTTGTGTGGGACGATCCAACATCAAGGAGTATTCTTGGAATTAAGACGGATGGTTTCATGGGCACACGAGA
TGAGGAGACCCGCAGATTTTTCAAGCACTCTTCAGTTCAAGTATTGCTTTGCCCACGATCTGCTGGGAAACGGCACAGCTGGGTGAAACAGCAGGAAAC
AGGAACCATTTTTACTCATCATCAGAAAACAGTTATTTTGGATGCTGATGCTGGCAATCATAAGAGGAAAATAATTGCTTTTGTTGGTGGCCTCGATTT
ATGCGGTGGGCGATATGATACACCTAGCCATCCTCTATTTAGGTCTCTTCAGACTGTGCACAAGGAGGATTATTACAATCCAAATTTTGCTACAGTTGA
TGCTCGTGGCCCAAGGGAACCATGGCACGACTTGCATTCCAAGATTGATGGTCCAGCGGCATATGATGTTTTGCAAAATTTTCAGGAGCGTTGGTTAAA
GGCATCCAAACGCCATGGGATTAAAAAGTTAGGAAAATCATATGATGATGCCCTCCTCAGTATTGAAAGAATACCTGATTTTATAAGCATTAATGACGC
AATATATTTTAGTGACAATGATCCAGAGACGTGGCATGTTCAGGTGTTTCGTTCTATTGATTCAAATTCTGCCAAAGGATTTCCAAAGGATCCACGGGA
AGCAACCCGCAAGAATCTTGTTTGTGGGAAGAATGTACTGATTGATATGAGCATACATACTGCATATGTCAATGCTATTCGTGGAGCGCAGCACTTCAT
TTATATTGAGAACCAGTATTTCATTGGATCTTCGTTTAATTGGGATTCAAACAAAGATATTGGGGCTAATAATTTAATACCAATCGAAATTGCTCTCAA
AATTGCAAACAAGATAAAGGCAAAAGAGAGATTCTCTGCATATATAGTAATTCCCATGTGGCCTGAGGGTAATCCAACAGGTGCTCCTACACAAAGAAT
TCTTTACTGGCAGCACAAGACAATGCAAATGATGTACGAAACAATATATAGGGCCCTGAAAGAAGAGGGTCTAGATGATCTATATGAGCCTCAGGATTA
TCTGAACTTCTTTTGTCTTGGCAATCGTGAAGTTGCTGACAGTCCAAGCACTTCAAACTCAACAAGCACTCCTCAGGAACAAGCTAGGAAACATAGAAG
ATTCATGGTATATGTACATTCAAAAGGCATGATTGTGGATGATGAATATGTGATAATTGGATCAGCTAATATCAATCAGAGGTCCATGGAAGGTATCAG
AGATACTGAGATTGCGATGGGTGCGTATCAGCCACAGTATACATGGGCAAGTAAGGTTTCTGCACCCCGTGGACAGATTTATGGGTACAGAATGTCTCT
TTGGGCGGAGCATATTGGAGTGGTTGAGGAAGGCTTCAACTATCCAGAGACCATGGAATGCATGAGACGAGTTCGCCAAATCGGTGAACAGAACTGGGA
ACGATTTGTTGATAATGAGGTGACTGAGATGAGAGGCCACCTAATGAAGTACCCTGTAAGTGTTGACCGTAAGGGCAAGGTGAAACCCTTGCCAGGATG
TACGTCATTCCCAGACATGGGTGGTAACATCTGTGGATCATTCCGAGCCATCCAAGAAAACCTCACAATA**TGA**

Supplementary Figure 2 A: Effect of 'C' insertion mutation in the OsPLDβ1 locus leading to a Premature Stop Codon Rice *OsPLDβ1* gene encodes for a 1046 amino acid polypeptide. *Ospldβ1-1* (*Os OsPLDβ1-KO1*) having 'C' nucleotide insertion leads to introduction of premature stop codon at 131 amino acid position with altered amino acid sequence (turquoise color) from 23 position (Karippadakam et al., 2026)

Classification: Personal

ATGGAGGGCGGAAACCACGGTGGGGGAGGGTACCCCTACCCGCCGCCGCAGCAGTATCCTTACCGTACGGGCAGTACCCATACCAGTATCCTCCACCGC
M E G G N H G G G G Y P Y P P P Q Q Y P Y R T G S T H T S I L H R

AGCAGCAGCCGCCGCCGCCCTCCGCCTACCTCTCGCCGTCCCGTTCGTTCCACGGTTACCCCTCCGCGCCGCCGCCGCAGCCGCAGCCGCAGCCGTACG
S S S R R R P P P T S R R P V R S T V T P P R R R R S R S R S R T

CGCACCATTCCGCCCCGCTCCAGCCTTACCCGCCGCCGCCGCAGCACCATGCCTACCCGCCGCCGCAGCCGCATCCGCCCTCGCCGTACGTCTACGACC
R T I P P R S S L T R R R R S T M P T R R R S R I R P R R T S T T

CTTACCACGCGCCCGCCGCGGCGTACCCTTCCTACCCGAGCCCCAACCCCAGCCCGTCCATCTCCCCGAGCTCCAGCTTCCACCACCACCCTGAACCGC
L T T R P P R R T L P T R A P T P A R P S P R A P A S T T T L N R

CATCGCCATCGCCCTCCGCGCCGTCGTACCCCTCCATTGCGGATGGGCTCGCCAACATGCACGTCTCCGACCGCCACGACTACCCGCCGCCGCCGTCGC
H R H R P P R R R T P P L R M G S P T C T S P T A T T T R R R R R

CGGCGGCCGTGCCCGCGGCGTCGTCCCCCTCCGTGCTCCCGCCTTCCGCGTCGTTCCCCGGCGGCGGGAGCAGCCACGGCGGTGGCGGGATGCAGATGG
R R P C P R R R P P P C S R L P R R S P A A G A A T A V A G C R W

TGCCGTACGGCCCTCCTGCCGGAGGGTCGCAGCACGGTGGGATGCAGATGGTGGCGTACGGCTCGCCGGCCGGAGGGTCGCAGCACGGCAGCGTGAGGC
C R T A L L P E G R S T V G C R W W R T A R R P E G R S T A A *

CATCCCTGAAGGTGGTGCTGCTCCACGGCACCCTCGACGTCTGGGTGTACGACGCTCGGAACCTCCCGAACAAGGATCTCTTCTCCAAGAGGGTCGGCG
ACCTCCTCGGTCCGCGCCTCATCGGCGCCGTCGGCAGCAAAATGTCCAGCGCCAACATGACCAGCGACCCCTATGTTACCATCCAGGTCTCCTATGCCA
CCGTCGCGCGCACCTACGTCGTTCCCAACAATGAGAACCCCGTTTGGACGCAGAATTTCCTTGTCCCTGTTGGGCATGATGCCGCCGAGGTCGAGTTCG
TTGTCAAGGACAATGATGTCTTCGGTGCTCAGCTCATAGGCACAGTGTCGATTCCTGCTGAGAAGCTCCTTTTTGGAGAAAGGATTAACGGCATCTACG
ATGTGCTTGAGTCCAATGGGAAGCCATGCGCTCAAGGTGCTGTGTTGCGGCTGTCCATACAGTACATCCCGGTGGCTCAGCTCAAAATGTATCACCATG
GTGTCATTGCCGGCCCAGATAGCCTGGGAGTTCCTAATACTTACTTCCCGATGCGGCGTGGCAATAGAGTGACACTGTATCAGGACGCACATGTGCCTG
ATGGTTGTCTCCCGGATTTCTGTCTTGATCACGGAATGCGCTATCAGCACGGGCAGTGTTGGCGTGATATCTACGATGCTATATGCCAAGCACGGCGGT
TGATTTACATTGTGGGATGGTCAGTGTTCCACACAATCCACCTCATCAGGGAAGGAGTTGAGAAGATGCCATCACTTGGGGAACTGTTGAAGATGAAGT
CACAGGAAGGGGTGAGGGTGTTGCTTCTTGTGTGGGACGATCCAACATCAAGGAGTATTCTTGGAATTAAGACGGATGGTTTCATGGGCACACGAGATG
AGGAGACCCGCAGATTTTTCAAGCACTCTTCAGTTCAAGTATTGCTTTGCCCACGATCTGCTGGGAAACGGCACAGCTGGGTGAAACAGCAGGAAACAG
GAACCATTTTTACTCATCATCAGAAAACAGTTATTTTGGATGCTGATGCTGGCAATCATAAGAGGAAAATAATTGCTTTTGTTGGTGGCCTCGATTTAT
GCGGTGGGCGATATGATACACCTAGCCATCCTCTATTTAGGTCTCTTCAGACTGTGCACAAGGAGGATTATTACAATCCAAATTTTGCTACAGTTGATG
CTCGTGGCCCAAGGGAACCATGGCACGACTTGCATTCCAAGATTGATGGTCCAGCGGCATATGATGTTTTGCAAAATTTTCAGGAGCGTTGGTTAAAGG
CATCCAAACGCCATGGGATTAAAAAGTTAGGAAAATCATATGATGATGCCCTCCTCAGTATTGAAAGAATACCTGATTTTATAAGCATTAATGACGCAA
TATATTTTAGTGACAATGATCCAGAGACGTGGCATGTTCAGGTGTTTCGTTCTATTGATTCAAATTCTGCCAAAGGATTTCCAAAGGATCCACGGGAAG
CAACCCGCAAGAATCTTGTTTGTGGGAAGAATGTACTGATTGATATGAGCATACATACTGCATATGTCAATGCTATTCGTGGAGCGCAGCACTTCATTT
ATATTGAGAACCAGTATTTCATTGGATCTTCGTTTAATTGGGATTCAAACAAAGATATTGGGGCTAATAATTTAATACCAATCGAAATTGCTCTCAAAA
TTGCAAACAAGATAAAGGCAAAAGAGAGATTCTCTGCATATATAGTAATTCCCATGTGGCCTGAGGGTAATCCAACAGGTGCTCCTACACAAAGAATTC
TTTACTGGCAGCACAAGACAATGCAAATGATGTACGAAACAATATATAGGGCCCTGAAAGAAGAGGGTCTAGATGATCTATATGAGCCTCAGGATTATC
TGAACTTCTTTTGTCTTGGCAATCGTGAAGTTGCTGACAGTCCAAGCACTTCAAACTCAACAAGCACTCCTCAGGAACAAGCTAGGAAACATAGAAGAT
TCATGGTATATGTACATTCAAAAGGCATGATTGTGGATGATGAATATGTGATAATTGGATCAGCTAATATCAATCAGAGGTCCATGGAAGGTATCAGAG
ATACTGAGATTGCGATGGGTGCGTATCAGCCACAGTATACATGGGCAAGTAAGGTTTCTGCACCCCGTGGACAGATTTATGGGTACAGAATGTCTCTTT
GGGCGGAGCATATTGGAGTGGTTGAGGAAGGCTTCAACTATCCAGAGACCATGGAATGCATGAGACGAGTTCGCCAAATCGGTGAACAGAACTGGGAAC
GATTTGTTGATAATGAGGTGACTGAGATGAGAGGCCACCTAATGAAGTACCCTGTAAGTGTTGACCGTAAGGGCAAGGTGAAACCCTTGCCAGGATGTA
CGTCATTCCCAGACATGGGTGGTAACATCTGTGGATCATTCCGAGCCATCCAAGAAAACCTCACAATA**TGA**

Supplementary Figure 2 B: Effect of ‘C’ deletion mutation in the OsPLDβ1 locus leading to a Premature Stop Codon Rice *Os PLDβ1* gene encodes for a 1046 amino acid polypeptide. *Ospldβ1-2* (*Os OsPLDβ1-KO2*) having ‘C’ nucleotide deletion leads to introduction of premature stop codon at 229 amino acid position with altered amino acid sequence (turquoise color) from 22 position (Karippadakam et al., 2026).

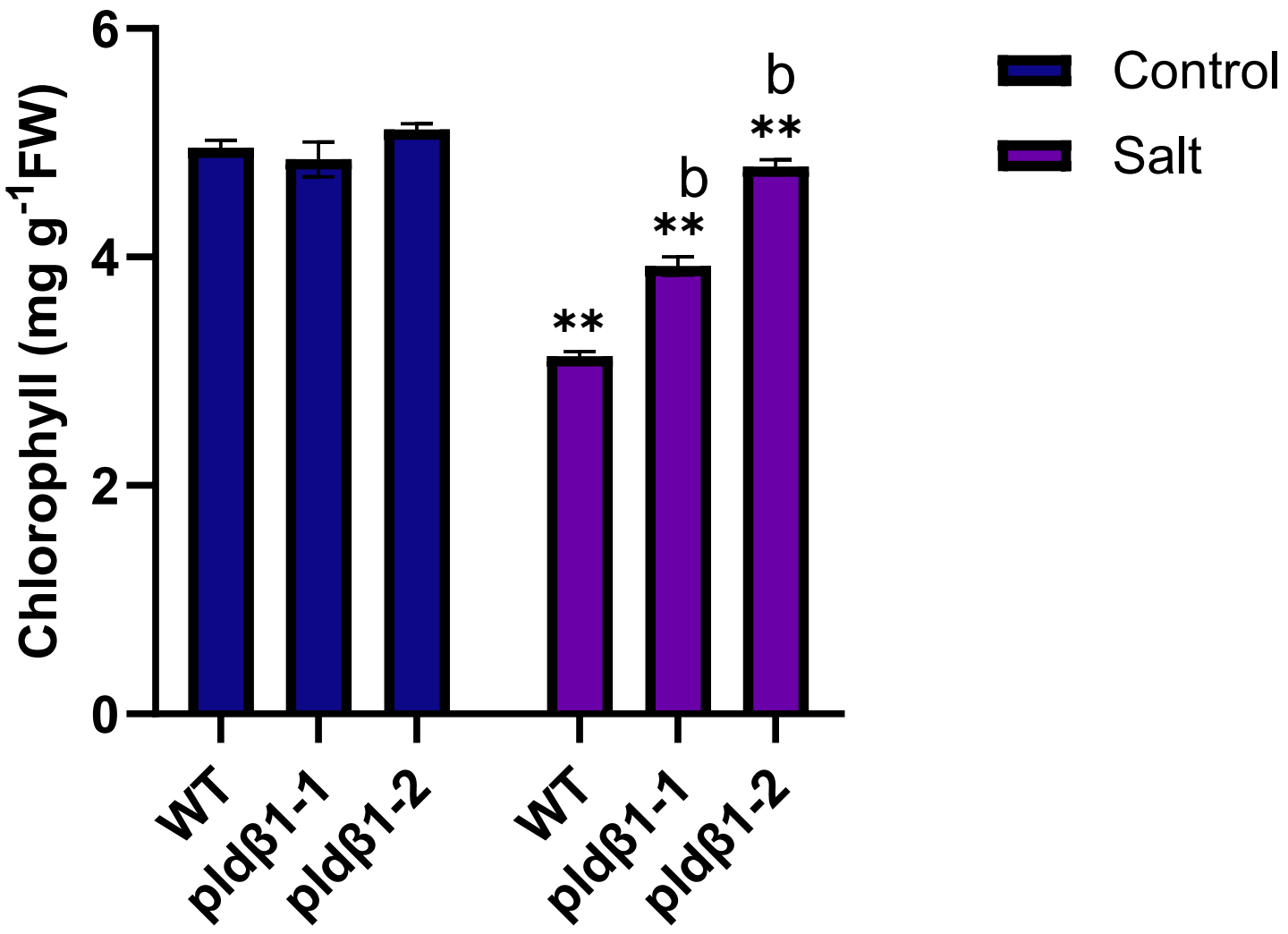

Supplementary Figure 3: Enhanced chlorophyll maintenance in mutant plants under salt stress. Chlorophyll content was measured in two-week-old wild-type (WT) and mutant leaves after 5 days of treatment with 0 (Control) and 150 mM NaCl. While salt stress reduced total chlorophyll in both genotypes, the mutant maintained significantly higher levels compared to WT. Values are means ± SD of three independent biological replicates. Letters and symbols indicate a significant difference between means ($p \leq 0.05$ or 0.01, one-way or two-way ANOVA Tukey HSD post hoc test).

Classification: Personal

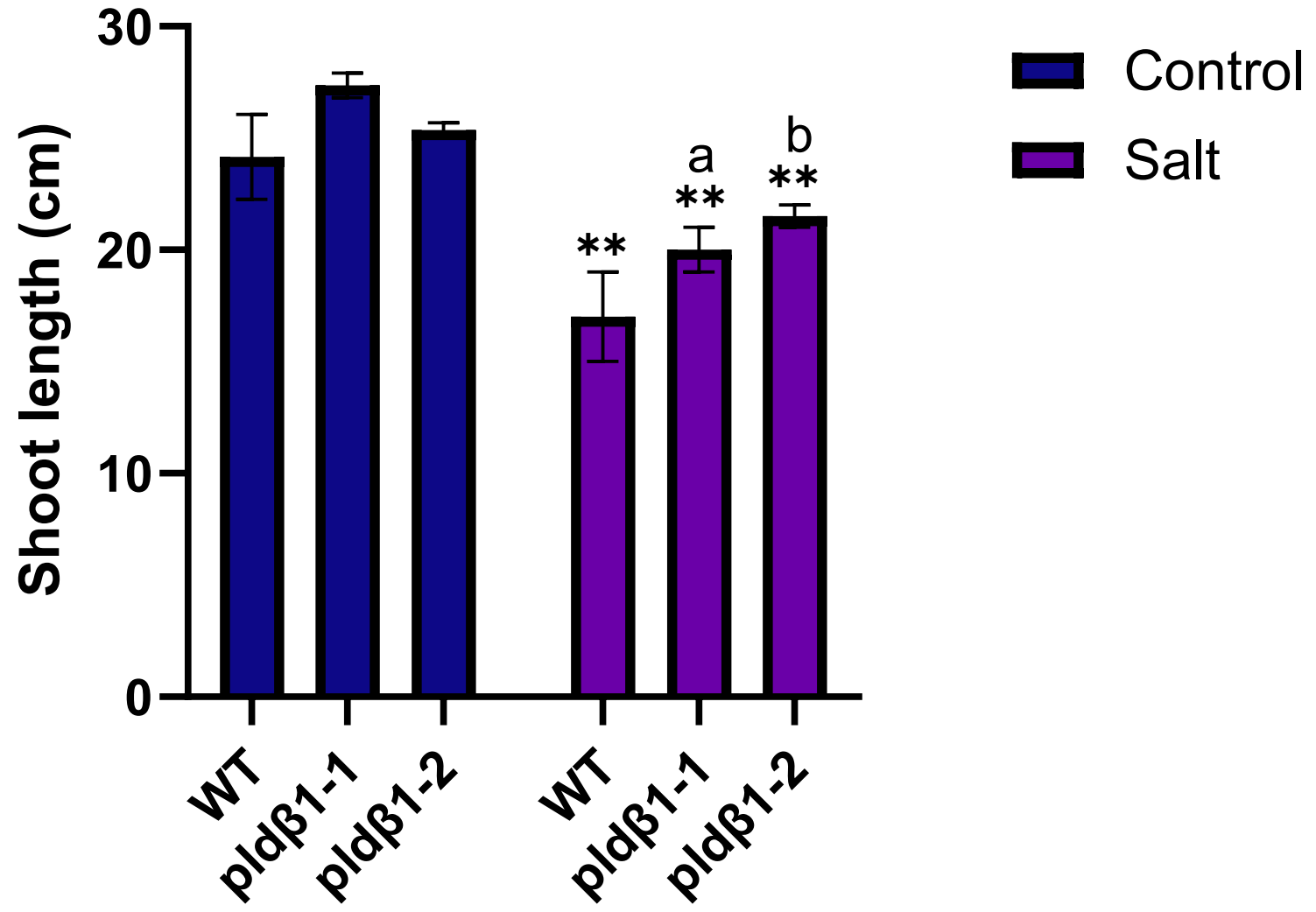

Supplementary figure 4: Effect of salinity on the shoot length of wild-type (WT) and mutant lines. Shoot length was measured from the root-shoot junction to the tip of the longest leaf. Shoot length measured in two-week-old wild-type (WT) and mutant leaves after 5 days of treatment with 0 (Control) and 150 mM NaCl Values are means ± SD of three independent biological replicates. Letters and symbols indicate a significant difference between means ($p \leq 0.05$ or 0.01, one-way or two-way ANOVA Tukey HSD post hoc test).

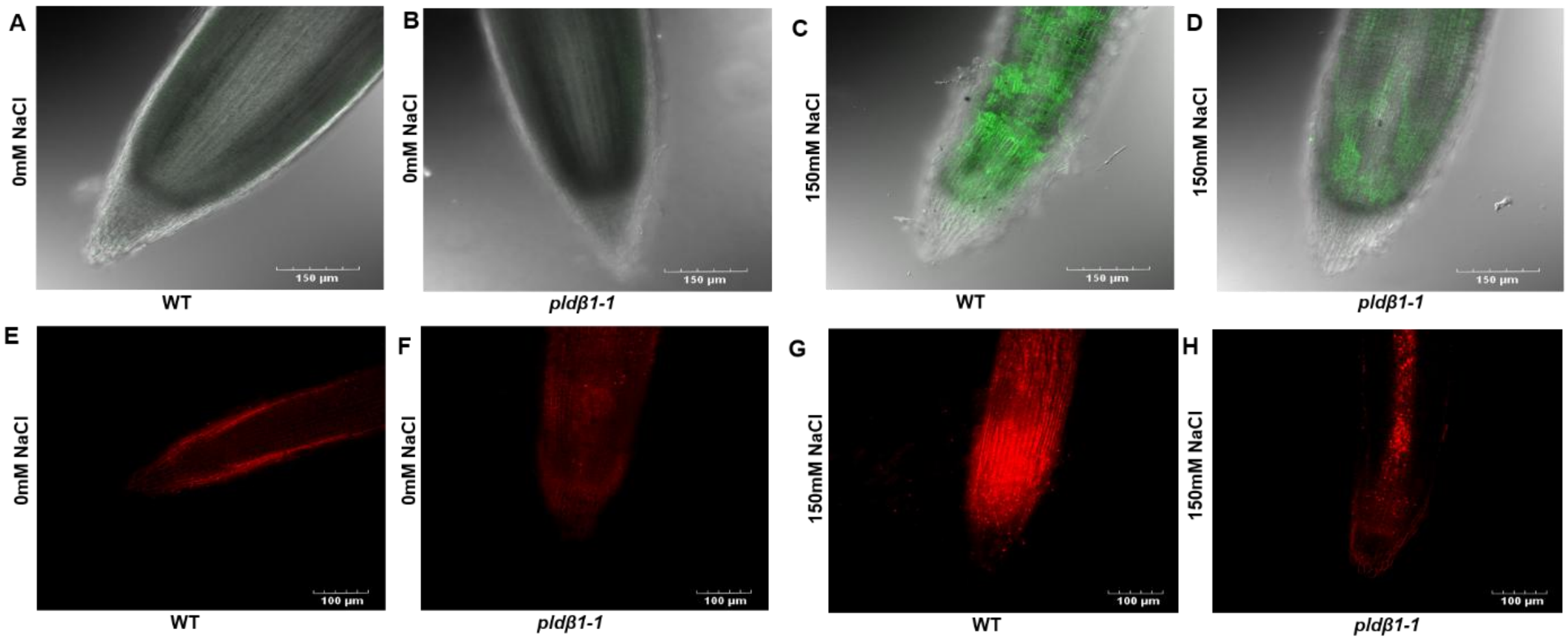

Supplementary figure 5: Visualization of Na^{+}-associated fluorescence and cell death in the roots of of WT and *Ospldβ1* rice lines. 1-week-old Seedlings of the WT and *Ospldβ1-1* lines were treated with 0mM and 150 mM NaCl for 48 h followed by staining with CoroNa green dye and propidium iodide (PI). Root tips were then visualized using confocal microscopy. Root tips of WT and *Ospldβ1-1* lines stained with CoroNa Green under control condition (A, B) and salt treatment (C, D). Red fluorescence associated with PI staining of the nuclei and cell wall-associated autofluorescence in the roots. Confocal image of PI-stained root tip under control (E, F) and salt stress condition (G, H).

Classification: Personal

Table S1. List of Primers used for real-time quantitative PCR (RT-qPCR) analysis.

Genes	5'------3'
OsUBIQ1-rtF	GGAGCTGCTGCTGTTCTAGG
OsUBIQ1-rtR	CACAATGAAACGGGACACGA
OsNHX1-rtF	ACATTGGAACGCTGGATGTA
OsNHX1-rtR	TCACAACACCTTCACCGAAT
OsTPC1-rtF	TTGGAAGGATGTTACGCTTG
OsTPC1-rtR	TTGGAAGGATGTTACGCTTG
OsHKT1,1-rtF	TTCACCACTCTTGCGGCTATG
OsHKT1,1-rtR	TGTTTGTAGCCAGTCTCCCCAG
OsHKT2,1-rtF	CACAGTCTCCTCGTTTGCGAA
OsHKT2,1-rtR	GCAAGAATCTGGCCGATGAA
OsHAK1-rtF	GTTGATGATGCTGATGTTGGAAG
OsHAK1-rtR	CCAACACTTTCAGCTGAAAC

Classification: Personal
